# Why we don’t always punish: Preferences for non-punitive responses to moral violations

**DOI:** 10.1038/s41598-019-49680-2

**Published:** 2019-09-13

**Authors:** Joseph Heffner, Oriel FeldmanHall

**Affiliations:** 1 Department of Cognitive, Linguistic, Psychological Sciences, Brown University, Providence, RI 02906 USA; 2 Carney Institute for Brain Science, Brown University, Providence, RI 02906 USA

**Keywords:** Reward, Human behaviour

## Abstract

While decades of research demonstrate that people punish unfair treatment, recent work illustrates that alternative, non-punitive responses may also be preferred. Across five studies (N = 1,010) we examine non-punitive methods for restoring justice. We find that in the wake of a fairness violation, compensation is preferred to punishment, and once maximal compensation is available, punishment is no longer the favored response. Furthermore, compensating the victim—as a method for restoring justice—also generalizes to judgments of more severe crimes: participants allocate more compensation to the victim as perceived severity of the crime increases. Why might someone refrain from punishing a perpetrator? We investigate one possible explanation, finding that punishment acts as a conduit for different moral signals depending on the social context in which it arises. When choosing partners for social exchange, there are stronger preferences for those who previously punished as third-party observers but not those who punished as victims. This is in part because third-parties are perceived as relatively more moral when they punish, while victims are not. Together, these findings demonstrate that non-punitive alternatives can act as effective avenues for restoring justice, while also highlighting that moral reputation hinges on whether punishment is enacted by victims or third-parties.

## Introduction

Punishment is pervasive throughout many societies^1^. Decades of research in behavioral economics and psychology empirically demonstrate that humans exhibit a strong desire to punish when deciding how to restore justice, even when it is costly^2^. However, more recent work reveals that if non-punitive alternatives are made available, punishment is not always systemically endorsed^3–5^. This suggests that preferences for punishment may be limited, perhaps to contexts where it is the only available option for restoring justice. Although the desirability of punishment has been questioned^6–8^, it remains unclear what features of a moral transgression motivate a punitive versus non-punitive response. Here, we explore this question, investigating when—and why—punishment is preferred as an instrument for restoring justice.

The desire to punish appears to lie either in its ability to change unjust behavior through deterrence^9^ or alleviate negative emotions triggered from being treated unfairly^10,11^. In either case, punishing a transgressor can reap positive benefits. There are, however, several negative consequences associated with responding punitively. For example, punishment can erode cooperation by turning prosocial cooperators into antisocial punishers^2,12,13^. In other cases, perceived unjustified punishment leads individuals to retaliate with retributive behavior, and this cycle of retribution collectively reduces the welfare of the group^14,15^. These findings together suggest that punishment may have mixed desirability depending on the context, and thus may not be a uniformly preferred method for restoring justice.

In contrast, non-punitive options have shown to be powerful motivators for restoring justice^16–18^. These non-punitive options typically address the victim’s needs through compensation (operationalized within the laboratory by endowing the victim with money) instead of focusing on punishing the perpetrator. Compensating the victim has many real-world analogues. For example, insurance companies pay reimbursement for stolen or damaged goods, and programs such as the *Office for Victims of Crime* allocate money (e.g., more than $2 billion in 2016) to help more than 5 million victims who have been impacted by crime^19^. Furthermore, monetarily rewarding good behavior—compared to sanctioning norm violators—appears to be one effective route for promoting cooperation^20–22^. Other research examining how rewards and sanctions differentially encourage cooperation reveals that rewards can produce better outcomes for the group compared to punishment alone^3^. Although these results suggest that, depending on the context, non-punitive measures may be viable alternatives, the boundary conditions (i.e., is there a tipping point for how much compensation is required before punishment is no longer desired?) remain unknown.

This inconsistent evidence naturally begs the question of *when* punishment is the preferred avenue for restoring justice. One possibility is that the decision to punish hinges on the context of the moral infraction^23^. One naturally-occurring context that may shape punishment preferences is when punishment is decided by a victim versus an impartial third-party^5,24^. In these cases, it is conceivable that different moral signals are generated depending on the perspective of the person deciding to punish, such that the act of punishing can either reap reputational benefits or damage one’s reputation. For example, when deciding to restore justice as a victim, punishment (such as gossiping about or publicly blaming norm violators, deciding to ostracize transgressors, etc.) may be perceived as retributive or vindictive while non-punitive responses may embody the doctrine of “turning the other cheek”, conveying that one values forgiveness^25^. If so, the reputational information gleaned from a victim deciding not to punish may signal that an individual has a positive moral character. In contrast, if a third-party member fails to punish, it may be interpreted as condoning the transgression, which would result in missing out on potential reputational advantages associated with punishing^26,27^. Simply put, punishment may act as a conduit for signaling different moral values depending on the context in which it arises and the perspective of the punisher.

To test these hypotheses and identify the social contexts in which preferences for punitive responses (as opposed to non-punitive alternatives such as compensation) are preferred, we leverage both behavioral economic games and crime vignettes. We fully parameterize the decision space to precisely measure the point at which people make trade-offs between punishment and compensation. If punishment is not a preferred method for restoring justice, then it should be less appealing as the victim’s needs are met through compensation. Indeed, it is possible that once the victim’s needs are sufficiently met, no amount of punishment—however small—will be preferred. In contrast, only when the victim’s monetary needs are not met should punishment become the preferred response. Additionally, we predict that the reputational value associated with decisions to punish will be modulated by the situation (i.e., deciding as a victim or a third-party). If punishment provides a greater positive reputational moral signal when deciding as a third-party, then third-party punishers should be preferred compared to punitive victims.

## Experiment 1: Compensating the Victim Abolishes Preferences to Punish Perpetrator

### Method

#### Participants

150 participants (48 women, 1 participant whose gender was unknown; age = 33.1 years, *SD* ± 10.6) were recruited through Amazon’s Mechanical Turk^28^ (AMT) to play a modified Justice Game^5^ (JG) and were paid $1.50 for completing the task, as well as a bonus determined from their decision on one randomly selected trial.

For all experiments: (1) participants played anonymously over the Internet and were not allowed to participate in more than one experimental session; (2) all studies were approved by Brown University’s Institutional Review Board; (3) all the methods were performed in accordance with the relevant guidelines and regulations of the institution, and informed consent of all participants was obtained; (4) we only analyzed the data of subjects who had unique Internet protocol addresses to avoid duplicate respondents; (5) all analyses were conducted in *R*^29^ and mixed-effects models were made with lme4^30^. (6) the desired sample sizes recruited for all studies were calculated using G*Power 3.1^31^. For all studies employing an economic game (Experiments 1, 2, and 4), our sample sizes were based on effect sizes (Cohen’s *d* = 0.28) observed in previous studies using a similar design^5^, which suggest than an approximate sample size of 110 participants (alpha = 0.05, beta = 0.90) was sufficient to detect preferences for compensation over punishment; (7) We collected a variety of post-experimental questionniares that were not analyzed for this manuscript. These include: Interpersonal Reactivity Index^32^, Social Value Orientation^33^, Delayed Discounting^34^, Intolerance of Uncertainty Scale^35^, and demographic data. For all studies we have reported all measures, manipulations, and participant exclusions. Finally, (8) we used Amazon’s Mechanical Turk to recruit all participants, whose subject pool has been shown to be representative of the broader population in the United States^36^.

#### Task

Participants played a modified version of a well-vetted economic paradigm known as the Justice Game^5,37^ (JG). In the JG, participants are paired with a unique anonymous player every trial and the two players must agree on how to split a sum of money. As the first mover, Player A is endowed with one dollar and proposes a division of this money with the participant, Player B (Player A: $1-x, Player B: x), in increments of $0.10 (prior work reveals incentive size—$1 versus $10—does not affect behavior^5^). We restrict offers from Player A (unbeknownst to participants, predetermined offers from a computer) to varying levels of unfairness, equally ranging from moderately unfair ($0.60, $0.40) to highly unfair splits ($0.90, $0.10). On each trial, participants, as Player B, respond by choosing between two options: (1) compensation: a non-punitive option where the victim deserves recompense—such that Player B is compensated some monetary amount while Player A’s payout remains unaffected; and (2) punishment: a ‘just deserts’ option where the perpetrator deserves punishment for the wrong committed—such that Player A is punished through monetary reduction while Player B is compensated^38^.

**Figure 1:**
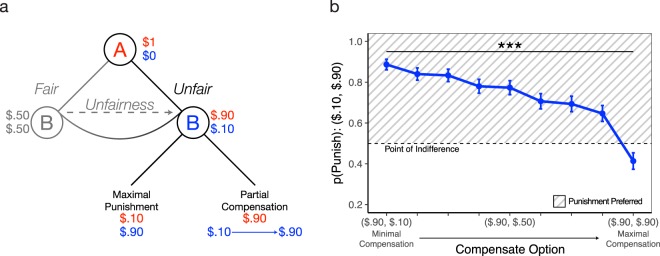
Partial Compensation Trials. (**a**) Game Tree. Player A (denoted in red) is endowed with one dollar and proposes a split with Player B. Participants, as Player B (denoted in blue), are informed of the proposed split and choose between two options to determine the final payouts of both players: (1) maximal punishment, an option which reverses the monetary payouts by fully reducing Player A’s payout (e.g., from $0.90 to $0.10) while also fully increasing the monetary payout for themselves (e.g., from $0.10 to $0.90), and (2) partial compensation, an option which partially increases the payout for themselves (varying on each trial) while Player A’s payout remains the same. **(b)** Results. We compute selection of maximal punishment juxtaposed against all partial compensation options. For example, the first data point shows a trial where participants chose between maximal punishment ($0.10, $0.90) and minimal compensation ($0.90, $0.10). Each data point represents a $0.10 increase in compensation. The point of indifference indicates a 50% chance of choosing punishment and the shaded area visualizes the area where punishment is the preferred option. Error bars reflect the standard error of the mean (SEM).

Accordingly, there are two trial types: partial compensation and partial punishment. In partial compensation trials, participants (Player B) must choose between maximal punishment to Player A and some amount of compensation to oneself (Fig. 1a). As an example, after receiving a highly unfair offer ($0.90, $0.10), the partial compensation option could increase Player B’s payout to some amount greater than $0.10, while not changing Player A’s payout ($0.90). This option is always pitted against maximal punishment, which fully reduces Player’s payout (to $0.10) and fully enhances Player B’s payout (to $0.90). In other words, participants choose between maximally punishing Player A at no cost to themselves, versus accepting some amount of compensation to avoid punishing Player A. Intuitively, when presented with these options, maximal punishment should be treated as the default, and thus this first experiment seeks to answer the question of how much compensation is required to shift people away from punishing?

Conversely, in partial punishment trials, Player B has the option to enhance their own payout while only partially reducing Player A’s payout. Effectively, they can choose to punish Player A for an unfair offer (partial punishment) or receive maximal compensation and forgo any punishment towards Player A. Critically, Player B’s payout is constant in these trials regardless of their choice and the only aspect that differs is how much punishment could be applied to Player A (ranging from minimal to maximal). This task structure allows us to determine the preference placed on applying punishment to Player A versus compensating oneself. Because partial punishment trials vary the amount of punishment available, the question is simply how much punishment is required (if any) to restore justice?

Participants completed 80 fully randomized, one-shot, self-paced trials: 20 partial compensation trials and 20 partial punishment trials deciding as the victim (Player B) and the same 40 trials deciding as a third-party (a within-subject design; see supplement for task instructions, full methodological details, and results of the third-party condition which show a similar pattern of behavior).

### Results

#### Preferences shift from punishing the perpetrator to compensating the victim

Given that punishment has been shown to be a highly preferred response to severe fairness violations^1^, we initially restricted our analysis to the most unfair offers ($0.90, $0.10). Using a logistic mixed-effects model, we tested our hypothesis based on previous work^5,37^ that increasing compensation for the self would result in less punitive behavior towards Player A (we report maximal models for all analyses^39,40^). In partial compensation trials with minimal compensation, participants unsurprisingly prefer maximal punishment since that option maximizes one’s own payout (Fig. 1b). However, participants become significantly less punitive as compensation to themselves increases ($$Odds\,Rati{o}_{compensation}$$ = 0.40; Table 1). In fact, once maximal compensation is available, the endorsement of punishment drops by almost 50 percentage points (Fig. 1b). This illustrates that participants’ preferences to punish a perpetrator attenuates as their own compensation increases. Replicating previous work^5,37^, once participants are fully compensated, punishment is no longer the most preferred response. This effect was observed for all fairness levels (see supplement).

**Table 1:** Greater compensation for victim leads to less punishment to perpetrator. $Punis{h}_{i,t}={{\rm{\beta }}}_{0}+$ ${{\rm{\beta }}}_{1}Compensatio{n}_{i,t}+{\rm{\varepsilon }}$.

Dependent variable	Estimate (SE)	z	p
**Punish**			
Intercept	7.97 (1.13)	7.04	<0.001***
Compensation	−0.91 (0.14)	−6.72	<0.001***

*Note*. Punish ~ Compensation, where Punish is coded as (1) if chosen, and elsewise 0. Compensation is indexed by participant and trial and is a continuous variable, ranging from minimal (Player B receives $0.10) to maximal (Player B receives $0.90). The model includes a random intercept and slope for compensation for each subject. ***p < 0.001.

#### Preferences to punish do not increase if there is ample compensation

**Figure 2:**
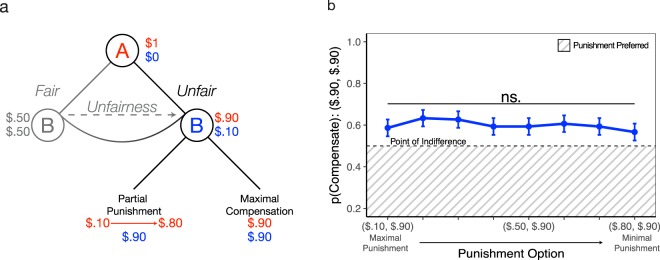
Partial Punishment Trials. (**a**) Game Tree. Player A is endowed with one dollar and proposes a split of that money with Player B. Participants, as Player B, are informed of the proposed split and choose between two options to determine the final payout of both players: (1) partial punishment, an option which reduces the monetary payout of Player A to some amount between $0.10 and $0.80 (varying on each trial), while also fully increasing the monetary payout for themselves, and (2) maximal compensation, an option where the payout to Player A remains the same ($0.90) but the payout to oneself is maximally increased ($0.90). **(b**) Results. We compute selection of maximal compensation juxtaposed against all partial punishment options. For example, the final data point on the right of the graph shows a trial where participants choose between maximal compensation ($0.90, $0.90) and minimal punishment ($0.80, $0.90). Each data point represents a $0.10 decrement from maximal punishment (Player A receives only $0.10) to minimal punishment (Player A receives $0.80). The point of indifference indicates a 50% chance of choosing punishment and the shaded area visualizes the area where punishment is the preferred option. Error bars are ± 1 SEM.

We next tested whether manipulating the degree of punishment in partial punishment trials can shift participants’ preferences in the wake of a fairness violation. That is, if a victim’s needs are already met through the maximal compensation option, how much punishment (if any) is endorsed? Contrary to the idea that there are appropriate amounts of punishment specific to certain degrees of infraction^38^ (i.e., a $0.90, $0.10 offer), we observed no increased desire to punish so long as participants received maximal compensation ($$Odds\,Rati{o}_{punishment}$$ = 0.91; Fig. 2b; Table 2). Simply put, given a specific infraction, participants were insensitive to the degree of punishment toward Player A, even in the face of highly unfair offers ($0.90, $0.10; see supplement for the results for other fairness levels, which all mirror the same behavioral pattern). These results indicate that the preference for punishment is non-dominant as long as one is sufficiently compensated, even when punishment is free and easy to implement.

**Table 2:** Minimal preference to punish if victim’s needs are met. $Compensat{e}_{i,t}={{\rm{\beta }}}_{0}+{{\rm{\beta }}}_{1}Punishmen{t}_{i,t}+\,{\rm{\varepsilon }}$.

Dependent variable	Estimate (SE)	z	p
**Compensate**			
Intercept	1.89 (0.72)	2.61	0.009**
Punishment	−0.10 (0.08)	−1.26	0.21

*Note*. Compensate ~ Punishment, where Compensate is coded as (1) if chosen, and elsewise 0. Punishment is indexed by participant and trial and is a continuous variable, ranging from minimal (Player A receives $0.80) to maximal (Player A receives $0.10). The model includes a random intercept and slope for punishment for each subject. **p < 0.01.

## Experiment 2: Compensation is Preferred in an Unconstrained Decision Space

Experiment 1 revealed that once one’s monetary needs are fully met, punishing Player A for making an unfair offer is no longer the dominant response. While Experiment 1 fully parameterized the possible punitive and compensatory decision space in the JG, it is possible that these observed behavioral patterns are an artifact of the forced choice design. If given an opportunity to freely express their own redistribution preferences, participants might demonstrate alternative behavioral patterns. Accordingly, we designed Experiment 2 to allow participants to express preferences for compensation and punishment independently by determining the final outcomes for both Player A and themselves (in $0.10 increments). Rather than pitting discrete choice pairs against one another, participants could choose to maximize (or minimize) punishment to Player A *and* maximize (or minimize) compensation to the self—or any combination in between. This experimental design does not force participants to select specific redistribution preferences operationalized by the researchers, and instead allows us to test whether—in an unconstrained decision space—participants naturally redistribute money in ways that increase compensation for the self and attenuate punishment towards the perpetrator.

### Method

#### Participants

200 participants (73 women, 1 participant whose gender was unknown; age = 32.8 years, *SD* ± 8.8) were recruited through Amazon’s Mechanical Turk. Participants were paid $0.50 for completing the task, as well as a bonus determined from their decision on one randomly selected trial.

#### Task

**Figure 3:**
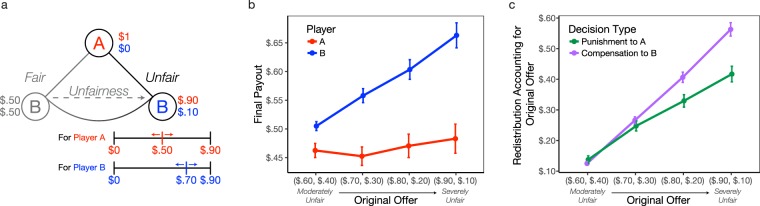
Unconstrained Redistribution Trials. (**a**) Game Tree. As before, Player A makes a split of one dollar with Player B. Participants, as Player B, determine the final monetary payouts using a visual analog scale (VAS). The monetary range of the VAS depends on the proposed offer, such that if Player A offered an unfair split ($0.90, $0.10) then the VAS would range from $0 to $0.90 for both players. This allows participants access to the full range of punitive and compensatory redistribution possibilities associated with each offer. The schematic shows an example where the participant chose to redistribute $0.50 to Player A and $0.70 to themselves after receiving an unfair offer ($0.90, $0.10). (**b**) Redistributions. Average monetary redistribution is plotted for Player A (in red) and Player B (in blue) as a function of the unfairness of the proposed offer. (**c**) Redistributions accounting for offer unfairness. The absolute difference between the final redistribution and original offer is plotted for compensation decisions (in purple) and punishment decisions (in green) as a function of the unfairness of the proposed offer. Error bars are ±1 SEM.

As in Experiment 1, participants play the Justice Game but this time they use a sliding visual analogue scale (VAS) to decide the final monetary outcomes of both Player A and themselves (Player B). The monetary amounts on the VAS depend on the fairness of the original offer, such that if Player A proposes an unfair split of ($0.90, $0.10) then the VAS’s maximum endpoint would be $0.90 (see Fig. 3a). This design ensures that the redistribution space is fully indexed according to the original offer from Player A. Participants can keep the proposed split the same (e.g., $0.90 for Player A, $0.10 for themselves), or, decrease or increase Player A’s payout as well as their own payout without restriction. Participants are explicitly told that the total monetary amounts of both players do not have to add up to one dollar.

Participants completed four one-shot, self-paced trials as the victim in response to offers ranging from relatively fair ($0.60, $0.40) to highly unfair ($0.90, $0.10). They also completed four trials as a third-party member (see supplement for task instructions, full methodological details, and results of the third-party condition). Trials were fully randomized.

### Results

Examining decisions to re-allocate money across all fairness infractions reveals that participants consistently redistributed approximately $0.50 to Player A (Fig. 3b; Table S5), which can be perceived as a form of minimal punishment (compared to taking away all of their money). However, when taking the unfairness of the original offer into account, we can calculate the amount of punishment toward Player A and compensation for oneself, by taking the absolute value of the final redistribution minus the original offer. This analysis reveals that participants increase both their own compensation and punishment toward Player A as unfairness increases (Cohen’s *d* = 1.21; Table 3). Critically, an interaction between decision type (compensation or punishment) and the unfairness of the original offer reveals that participants compensate themselves more relative to punishing Player A as unfairness increases (Cohen’s *d* = 0.50; Table 3; Fig. 3c). Together, these results reveal that while punishment does increase with unfairness, compensation for oneself as a means of justice restoration increases even more as a function of the violation.

**Table 3:** Participants compensate relatively more than punish as unfairness increases. *Redistribution Accounting for* $Offe{r}_{{\rm{i}},{\rm{t}}}={{\rm{\beta }}}_{0}+{{\rm{\beta }}}_{1}\,Decision\,Typ{e}_{{\rm{i}},{\rm{t}}}\times {{\rm{\beta }}}_{2}\,Original\,Offe{r}_{{\rm{i}},{\rm{t}}}+{\rm{\varepsilon }}$.

Dependent variable	Estimate (SE)	t	p
**Redistribution Accounting for Offer**			
Intercept	28.29 (1.69)	16.75	<0.001***
Decision Type	5.72 (1.59)	3.60	<0.001***
Original Offer	9.21 (0.44)	20.99	<0.001***
Decision Type × Original Offer	5.34 (0.62)	8.61	<0.001***

*Note*. Redistribution Accounting for Offer ~ Decision Type × Original Offer, where Redistribution Accounting for Offer is a continuous variable defined as the absolute value of the redistribution minus the original offer and is indexed in cents. Punishment serves as the reference (0) for Decision Type (punishment to A/compensation to B). Original Offer was mean centered before being entered into the regression. Decision Type and Original Offer are indexed by participant and trial. The model includes a random intercept and slope for decision type for each subject.

*** p < 0.001.

## Experiment 3: Preferences for Compensation Generalize to Severe Moral Transgressions

In Experiment 2, when participants were given full control over the final redistribution outcomes, we again found evidence that they preferred to compensate themselves more than punish the perpetrator. However, it remains unclear if these preferences exist only in contexts where there are relatively minor violations (i.e., unfair splits of one dollar in one-shot economic games), or if they generalize to other more egregious transgressions. In Experiment 3, we explore this question, investigating whether preferences to punish and compensate scale with increasingly severe moral transgressions. While past research illustrates that people endorse the doctrine of “proportional punishment” (such that punishment scales with the severity of the crime committed^38^), it remains unknown whether compensation to the victim also scales as moral violations become increasingly pernicious. Given our findings in Experiments 1–2 that compensation can rebalance the scales of justice, we posited that people should increasingly endorse compensation as transgressions become more severe.

### Method

#### Participants

271 participants (163 women; age = 32.8 years, *SD* ± 10.1) were recruited through Amazon’s Mechanical Turk. Our sample size for the data reported in the manuscript (responding as the victim, N = 134) was based on the large effect size of punishment judgments (Cohen’s *d* = 1.18) observed in previous studies using a similar design^38^. However, because it is unknown whether compensation judgments would have a similar effect size as punishment, we used a conservative medium effect size^41^ (Cohen’s *d* = 0.50;) which suggested that a sample size of *N* = 36 is sufficient to observe an effect of moral severity on compensation judgments with 90% power (α = 0.05). Participants were paid $2.50 for completing the task.

#### Task

To test whether judgments of punishment and compensation generalize to more severe moral violations, we developed a series of crime vignettes. We wanted naturalistic descriptions of crimes which contained enough details to justify judgments of moral condemnation without providing extraneous or nuanced aspects of each crime. Accordingly, we pulled crime examples from the “Uniform Crime Reporting Handbook” published by the Federal Bureau of Investigation^42^, a publication which establishes uniform definitions for crimes based on a hierarchical classification procedure. The crime vignettes ranged from disorderly conduct to murder (see supplement for all crime descriptions). Vignettes were selected and minimally altered to include one perpetrator and one victim, which ensured that judgments of punishment and compensation could be constrained to a single individual across all crimes.

Participants were presented with short vignettes of a variety of crimes and were asked to rate the degree of compensation for the victim and punishment for the perpetrator. For example, a crime involving robbery stated: “You were walking down the street when an assailant grabbed you and held a knife to your throat. The assailant removed your wallet from your pocket and ran.” After reading each vignette, participants answered three questions using a continuous Likert scale ranging from none (−50) to a lot (50): (1) “How much should you be compensated for the crime?” (*No compensation – a lot of compensation)*; (2) “How much should the perpetrator be punished for the crime?” (*No punishment – a lot of punishment)*; and (3) “Please rate the moral severity of the crime” (*Not severe – Very severe*). The ends of the scales were purposely left ambiguous (e.g., ‘a lot of punishment’), so that subjects were free to infer their own subjective idea of what punishment should be levied on the perpetrator. This allowed us to directly compare subjective rates of punishments against compensation, while also extending our findings from Experiments 1–2, which used objective punitive and compensatory responses (i.e., money).

Participants responded to 29 crime vignettes either from the perspective of the victim or from the perspective of a third-party observing the crime (a between-subject design). For the third-party condition, a crime involving robbery stated: “A man was walking down the street when an assailant grabbed him and held a knife to his throat. The assailant removed the victim’s wallet from his pocket and ran”. The order of trials was fully randomized (see supplement for task instructions, full methodological details, and results of the third-party condition).

### Results

**Figure 4:**
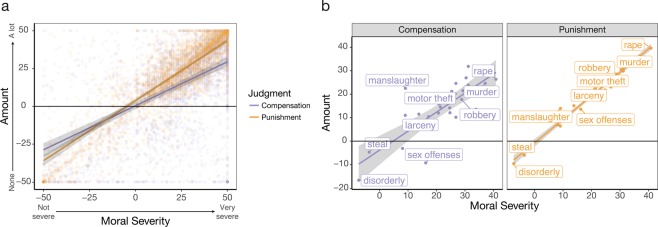
Crime Vignettes. (**a**) Results. Amount of compensation or punishment is plotted for compensation judgments (in purple) and punishment judgments (in orange) as a function of perceived moral severity. Plot lines reflect parameter fits based on trial-wise mixed-effects regressions. Shaded error bars reflect 95-percent confidence intervals. **(b**) Results grouped by crime. For each crime vignette, we averaged participants’ judgments of moral severity, compensation, and punishment. Average amount of compensation and punishment allotted is plotted as a function of the perceived moral severity of each crime. A subset of the 29 crimes are labeled.

To examine how participants’ punishment and compensation judgments varied across vignettes, we conducted a linear mixed-effects regression predicting amount of punishment or compensation as a function of judgment type (punishment or compensation) and degree of moral severity. Because participants likely would have different perceptions of the severity of the same crime, we included moral severity as a predictor. Results revealed that both compensation and punishment judgments scaled upwards as the crime became more morally severe (Cohen’s *d* = 3.47; Fig. 4a; Table 4). Participants gave harsher punishments to the perpetrator and more generous compensation to themselves (the hypothetical victim) as their perceived moral severity of the crime increased. A similar pattern was observed when participants responded as a third-party, see supplement for details.

**Table 4:** Compensation and punishment scale with moral severity. $Amoun{t}_{{\rm{i}},{\rm{t}}}={{\rm{\beta }}}_{0}+{{\rm{\beta }}}_{1}Moral\,severit{y}_{{\rm{i}},{\rm{t}}}\times$ ${{\rm{\beta }}}_{2}Judgmen{t}_{{\rm{i}},{\rm{t}}}+{\rm{\varepsilon }}$.

Dependent variable	Estimate (SE)	t	p
**Amount**			
Intercept	13.36 (1.14)	11.74	<0.001***
Moral severity	14.71 (0.80)	18.46	<0.001***
Judgment	8.14 (0.97)	8.36	<0.001***
Moral severity × Judgment	5.35 (0.66)	8.05	<0.001***

*Note*. Amount ~ Moral severity × Judgment, where Amount is a continuous variable ranging from −50 (no compensation/punishment) to 50 (a lot of compensation/punishment). Moral severity and judgment are indexed by participant and trial. Moral severity is a continuous variable ranging from −50 (not severe) to 50 (very severe) but standardized to improve convergence and interpretation of the model. Compensation serves as the reference (0) for Judgment. The model includes a random intercept and slopes for moral severity, judgment, and the interaction for each subject.

*p < 0.05. **p < 0.01. ***p < 0.001.

We further observed an interaction between the amount of punishment or compensation and moral severity such that participants increased the amount of punishment relative to compensation for more morally severe crimes (Cohen’s *d* = 1.56; Fig. 4a; Table 4). Aggregating ratings of moral severity for each vignette, participants judged crimes such as murder and forcible rape as the most severe and thus allocated the most punishment and compensation for these crimes (Fig. 4b). While these results indicate that, at least in hypothetical judgments, participants increase punishment and compensation as crime severity increases (and prefer punishment more than compensation for the most egregious crimes), it also reveals that compensation is a desirable method for restoring justice for crimes that are not too severe (e.g., disorderly conduct or stolen property). Overall, these results suggest that the preference to compensate the victim generalizes to moral transgressions observed in the real world, and both compensatory and punitive preferences increase as the crime increases in moral severity.

## Experiment 4: Non-punitive Responses can Confer Positive Moral Reputation

In Experiment 3, when participants were asked to imagine being the victim in a series of crime vignettes, we found evidence that participants compensate themselves for both minor and severe moral violations. However, pressing questions remain; namely, why are decisions to compensate sometimes the preferred option for restoring justice, especially when punishment is free and easy to enact? We explore this question in Experiment 4, investigating whether decisions to punish play a different role in signaling a set of moral values depending on the perspective of the person deciding how to restore justice. Specifically, we predict that victims who chose the non-punitive, prosocial redistribution option will be associated with a positive moral signal because it will be akin to “turning the other cheek.” In contrast, third-party members who fail to punish will be perceived as condoning the transgression. Thus, the punitive option should only be associated with a positive moral signal when deciding as a third-party.

### Method

#### Participants

200 participants (84 women, 4 participants whose gender was unknown; age = 34.9 years, *SD* ± 10.5) were recruited through Amazon’s Mechanical Turk to play a new variant of the JG and two partner selection tasks. Participants were paid $2.50 for completing the task, as well as a bonus determined from their decision on one randomly selected trial.

#### Task

**Figure 5:**
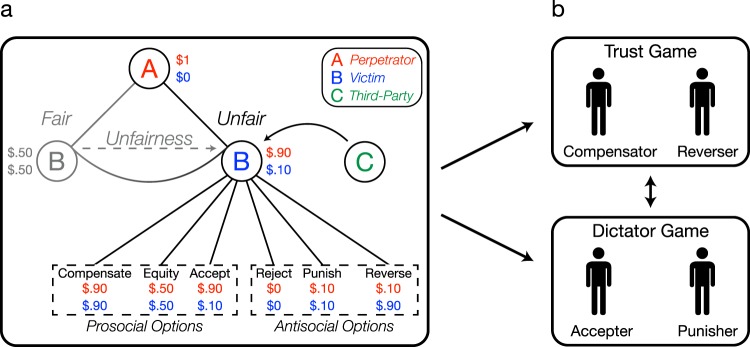
Two-Stage Economic Game. (**a**) Justice Game Phase. Player A makes a split of one dollar with Player B. Participants play the game either as Player B or Player C (between-subjects design). When playing as Player C, participants are a third-party who decide on behalf of an anonymous Player B such that the final payouts are for Player A and Player B. On each trial, participants pick between two randomly presented options from the six available options. The game is constructed such that there are three prosocial options (Compensate, Equity, and Accept) and three antisocial options (Reject, Punish, and Reverse). Participants are told that other participants (their future partners) also completed this task. (**b**) Partner Selection Phase. Participants are instructed to select a partner for the Trust Game (TG) and the Dictator Game (DG; order counterbalanced). Participants make a forced choice between two partners, labeled by their most endorsed option in the Justice Game Phase. Here, we show an example trial in the TG where one potential partner most endorsed Compensate (i.e., ‘Compensator’) in the JG and the other potential partner most endorsed Reverse (i.e., ‘Reverser’). In the DG example, an ‘Accepter’ is pitted against a ‘Punisher’. Participants completed all possible forced choice pairings (15 trials) for both the TG and DG.

Participants played a two-stage economic game, which began with a variant of the JG. In this version of the JG, after Player A makes an offer, Player B can reapportion the money by choosing between two randomly selected options drawn from the following six options (Fig. 5a): (1) Accept: agree to the proposed split ($1-x, x); (2) Equity: split the monetary pie equally so that both players receive half of the initial endowment ($0.50, $0.50); (3) Compensate: increase Player B’s own payout to equal Player A’s payout, thus enlarging the pie to maximize both players’ monetary outcomes ($1-x, $1-x); (4) Punish: reduce Player A’s payout to the original amount offered to Player B (x, x); (5): Reverse: reverse the proposed split, the ‘just deserts’ motive where Player A deserves punishment proportional to the unfairness of the offer; and finally, (6): Reject: both players receive nothing ($0, $0). The first three options can be considered more prosocial in nature, with little or no punishment of Player A; while the last three options can be considered more antisocial in nature, where punishment acts as a key ingredient for restoring justice. By adding the Reject option (which was not available in the original JG version^5^), we ensure that there is an equal probability of being presented with a prosocial option (Accept, Equity, and Compensate) or antisocial option (Punish, Reverse, and Reject). In other words, because Player A does not know which two options will be given on any given trial, having an equal number of prosocial and antisocial options buffers against any interpretation that Player A could be strategically giving unfair offers. Here, we use these labels—prosocial and antisocial—to differentiate options not involving punishment versus those that do (although these labels were never presented to the subjects). Participants are only given two options on any trial, such that each option is randomly paired with one alternative option, resulting in every combination pair for a total of 15 unique pairs. In this first phase of the game, we have participants play the JG so they can learn all the psychological motivations behind each redistribution option.

In a subsequent partner selection phase (Fig. 5b), participants are informed they will be choosing their partner for two future economic games (unknown to participants, the experiment concludes after the partner selection phase ends). The only information participants are given to inform their choice is their potential partners’ past behavior in the JG. For example, participants might be informed that a partner’s most endorsed option was “Reverse”, while another partner preferred “Equity”. Unlike the previous experiments, here, we label each of the options (i.e., Accept, Equity, Compensate, Punish, Reverse, and Reject) to reduce the cognitive load on having to infer the option name from monetary redistributions. Using a between-subjects design, participants either completed the two-stage economic game from the perspective of the victim, in which participants play the game as Player B, or from the perspective of an impartial third-party, termed Player C. When playing as Player C, participants are asked to make decisions on behalf of an anonymous Player B. Unlike decisions as the victim, third-party decisions are non-costly and non-beneficial since the final choice only influences the monetary outcomes of Players A and B. The critical test is whether those who punished would be selected as a social partner at the same rate regardless if they made the decision as a victim or a third-party.

In addition, we were interested in examining the boundary conditions for when punishment would be perceived as a positive moral signal. We posited that third-party punishers may not always be systematically preferred as social partners, and that this preference will depend on the demands of the social situation. Accordingly, participants were tasked with selecting partners for both the Trust Game^43^ (TG) and the Dictator Game^44^ (DG). For the Trust Game, participants were selecting partners who would be the second mover, which indexes the perceived trustworthiness of their partner. When selecting partners for the Dictator Game, participants chose partners who would be the first mover, which indexes the perceived generosity of their partner. We chose these two games on the assumption that the reputational benefits associated with punishing might shift between these different social contexts. Take for example previous work illustrating that third-party decisions to punish are taken as a positive signal of one’s trustworthiness^23^: those who are willing to punish norm violators are seen as more likely to observe the norm of reciprocity (i.e., reciprocating trust) in the Trust Game.

There may be other social contexts, however, in which punishing is not perceived as a desirable behavior. It is possible that punitive partners would not be preferred in contexts that require generosity or altruism because the original punishment may construed as spiteful^45^. Rather, someone who behaved prosocially in the Justice Game—for example, by equally redistributing the money or compensating without punishing—would be more highly sought after than someone who punished the perpetrator. To test this, we utilized a similar pairwise comparison design used in Experiment 1 and fully parameterized the space by comparing each partner type against all others. All participants completed 15 self-paced partner selection trials for the TG and 15 self-paced partner selection trials for the DG, and the order of these games was counterbalanced.

### Results

#### When deciding as a victim, punishment is a poor signal of trustworthiness

**Figure 6:**
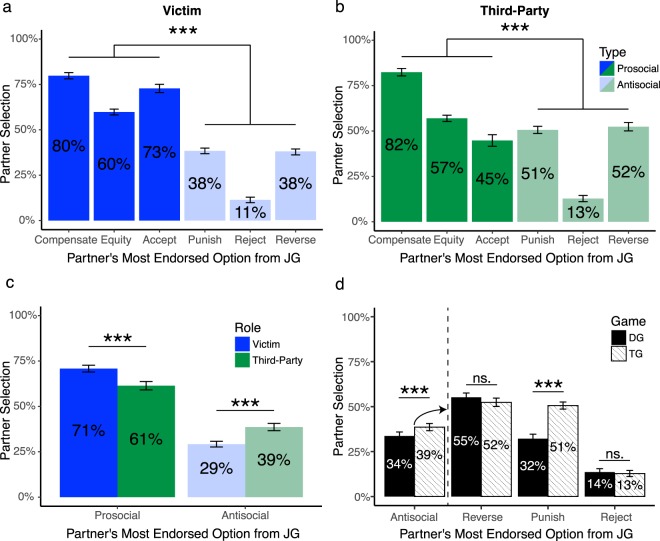
Prosocial partners are preferred over antisocial partners. Partner preference is computed as the frequency an option is selected from all available trials, such that each option’s endorsement rate is out of 100%. (**a**) Partner selection for the TG when responding as the victim. (**b**) Partner selection for the TG when responding as a third-party. (**c**) Partner selection for the TG collapsed across prosocial and antisocial responses as victim and third-party. (**d**) Preference for partners who selected one of the antisocial options in the JG (punish, reverse, or reject) for the Trust Game (TG) compared to Dictator Game (DG) when responding as a third-party (see supplement for all data). Error bars are ± 1 SEM.

We compute partner selection by the number of times a partner who preferred a particular option (e.g., a Compensator) is chosen for a Trust Game compared to every possible alternative partner (i.e., Accepter, Equity Maximizer, Punisher, Reverser, and Rejecter). Participants showed a strong preference for engaging with victims who responded prosocially (i.e., selected Accept, Equity, or Compensate options, compared to the other three antisocial partners; Friedman’s test, χ^2^(1) = 88.36, *p* < 0.001, Wilcoxon Signed-Rank test post-hoc effect size r = 0.93; Fig. 6a), with an especially strong desire for engaging with those who Compensated in the wake of a fairness violation (80%). In contrast, participants least preferred to play with partners who Rejected offers as a victim (11%). These findings offer evidence that choosing prosocial actions as a victim confers strong reputational benefits and offer one possible explanation for why non-punitive decisions may be preferred.

#### When deciding as a third-party, punishment is a relatively better signal of trustworthiness

Although participants still showed an overall preference for third-party Compensators (82%), when directly comparing preferences for partners who responded antisocially (collapsing Punish, Reverse and Reject options) as the victim versus as a third-party, we observed a stark difference in how punitive responses shaped subsequent engagement (Fig. 6c). Partners who selected the antisocial, punitive option as a third-party were more likely to be selected as partners for a Trust Game than those who responded punitively as the victim (Welch’s *t*-test, *t*(147.32) = 6.98, *p* < 0.001, g = 0.98). Interestingly, there was a steep and significant reduction in preferring third-parties who ‘Accepted’ an unfair offer on behalf of another (45%; Fig. 5b) compared to victims who did the same (73%; Fig. 5a: Kruskal Wallis test, χ^2^(1) = 38.54, *p* < 0.001, r = −0.44). While there is a dominant preference for prosocial third-party partners who compensate, these findings suggest that punishment can send a relatively better moral signal of one’s trustworthiness when that decision is enacted by a third-party compared to a victim.

#### Context modulates perception of third-party punishment

We next examined whether the reputational benefit for third-party punishment is stable across different social contexts. While a similar overall preference for prosocial over antisocial partners was also observed in contexts valuing generosity (i.e., in the Dictator Game; see supplement for DG results), there were significant differences in how frequently antisocial third-parties were selected in the TG compared to the DG. Third-parties who chose either to Punish, Reverse, or Reject in the JG (the antisocial options) were less preferred as partners in the DG compared to the TG (paired *t*-test on combined data from all three antisocial partners, *t*(99) = −4.47, *p* < 0.001, g = −0.38; Fig. 6d). When examining each option separately, Bonferroni-corrected post-hoc analysis showed that only third-parties who punished were significantly less preferred in the DG than the TG (paired t-test, t(99) = −8.11, p < 0.001, g = −0.82; Fig. 6d), and there were no contextual differences for those who chose Reverse or Reject. These results reveal that the reputational value of third-party punishment is not stable but shaped by the context of the situation. In other words, there is a significant decrease in preferring punitive partners when the situation values altruistic tendencies (i.e., generous offers in the DG), suggesting that punishment is not always interpreted as a signal of good moral character. In contexts which have strong social norms of reciprocity, such as the TG, it is possible that those who punish send a signal that they would be willing to uphold and enforce these norms.

## Experiment 5: Lenient Victims and Punitive Third-Parties are Perceived as Moral

Experiment 4 demonstrated that victims who punish are not preferred as social exchange partners, especially compared to victims who refrain from punishing. For those who do decide to punish, third-parties are preferred to victims. However, it is unclear whether third-parties and victims who punish are actually perceived as more or less moral, respectively. Accordingly, we ran a final experiment to probe whether those who punish as third-parties and those who refrain from punishing as victims are perceived as moral. In contrast, we posited that third-parties who fail to punish and victims who desire punishment should be perceived as similarly immoral.

### Method

#### Participants

189 participants (117 women; age = 31.3 years, *SD* ± 10.2) were recruited through Amazon’s Mechanical Turk. Participants were paid $0.50 for completing the task. Our sample size was based on the effect size (Cohen’s *d* = 0.26) observed in previous studies examining third-party punishment^5^, which indicated that a sample size of *N* = 129 is sufficient to observe this effect with 90% power (α = 0.05).

#### Task

Similar to Experiment 3, we used a crime vignette to test the reputational effects of a victim or third-party deciding to punish (or not punish) a perpetrator. We adapted a fictional case about vigilantism which details a shoplifting crime^46^. Using a 2 × 2 between-subjects design, participants read one of the following four vignettes: (1) the victim (store owner) monetarily punished the perpetrator for shoplifting; (2) the victim chooses not to punish; (3) a third-party (judge) monetarily punishes the perpetrator; or (4) the third-party chooses not to punish (see supplement for full descriptions of each vignette). Participants only made a single judgment where they rated the moral character of the victim or third-party, ranging from −50 (*Immoral*) to 50 (*Moral*) on a continuous Likert scale.

### Results

**Figure 7:**
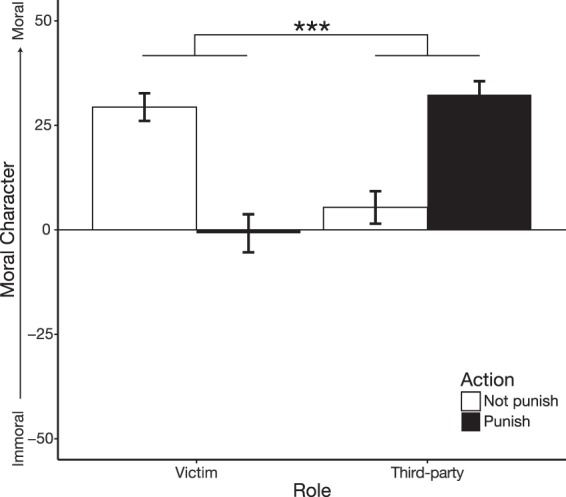
Perceptions of the punisher’s moral character depends on their role. Mean moral character judgments as a function of role (victim or third-party judge) and action (punish or not punish). Moral character ranges from −50 (Immoral) to 50 (Moral). Error bars are ±1 SEM.

To examine how participants’ perceptions of moral character differed as a function of who was deciding to punish, we conducted a linear regression where moral character is predicted by role (victim/third-party) and action (punish/did not punish). Results reveal an interaction between role and action: A judge who punishes is perceived to be significantly more moral compared to a victim who punishes, and victims who refrain from punishing are perceived as significantly morally superior to judges who fail to punish (Cohen’s *d* = 0.60; Fig. 7; Table 5). Both judges who do not punish and victims who do punish are perceived as having neutral moral characters (statistically not different from 0 = ’neutral moral character’, one sample t-test against 0 for third-party: *t*(48) = 1.39, *p* = 0.17; for victim: *t*(47) = −0.18, *p* = 0.86). Furthermore, judges who punish and victims who refrained from punishing—both of whom were perceived to be high in moral character—were indistinguishable from one another (Welch’s *t*-test, *t*(92.26) = 1.04, *p* = 0.30, g = 0.21). These results accord with the hypothesis that the perception of one’s moral character hinges on whether they are deciding as a victim or third-party: a victim who turns the other cheek, when they could alternatively enact vigilante justice, signals a positive moral character, while a judge gets a positive moral boost by punishing.

**Table 5:** Moral character of punisher depends on role. $Moral\,characte{r}_{{\rm{i}},{\rm{t}}}={{\rm{\beta }}}_{0}+{{\rm{\beta }}}_{1}Rol{e}_{{\rm{i}},{\rm{t}}}\times {{\rm{\beta }}}_{2}Actio{n}_{{\rm{i}},{\rm{t}}}+{\rm{\varepsilon }}$.

Dependent variable	Estimate (SE)	t	p
**Moral character**			
Intercept	32.35 (3.99)	8.10	<0.001***
Role	−33.18 (5.50)	−6.04	<0.001***
Action	−26.98 (5.47)	−4.93	<0.001***
Role × Action	57.18 (7.63)	7.49	<0.001***

*Note*. Moral character ~ Role × Action, where Moral character is a continuous variable ranging from −50 (Immoral) to 50 (Moral). Role and Action are between-subject variables and indexed by participant and trial. Third-party serves as the reference (0) for Role and Punish serves as the reference (0) for Action.

***p < 0.001.

### Open practices

 The data and analysis scripts that support the findings of these studies are avaliable online at the Open Science Framework at the following URL: https://osf.io/ft9bv/ 

## Discussion

Until now, it was unknown *when*—and under what conditions—people preferred punishment as a means of restoring justice. Our findings are twofold. First, dovetailing with previous work^5,37^ we demonstrated that once a victim’s needs are sufficiently met through monetary compensation, there is little desire to punish (Experiment 1). Even in a non-constrained decision space, participants freely decide to compensate themselves and administer relatively low amounts of punishment to the perpetrator—despite being able to fully reduce the perpetrator’s payout (Experiment 2). Furthermore, preferences for compensation appear to be a viable method of justice restoration for even the most egregious crimes, and is equally preferred to punishment when the infraction is not too severe (Experiment 3). Second, we observed that the reputational benefits associated with non-punitive and punitive measures are modulated by the perspective of the deciding agent (Experiment 4). Choosing not to punish as a victim sends a positive moral signal that one is likely to be both trustworthy and altruistic. In contrast, punitive responses are preferred when made by a third-party compared to similar punitive responses made by a victim. However, punishing as a third-party is not ubiquitously perceived as a positive moral signal: when a social situation emphasizes generosity, preference for third-party punishment declines. Moreover, lenient victims and punitive third-parties are perceived as having similar positive moral character, while third-parties who fail to punish are perceived as being more immoral (Experiment 5). Together, these results elucidate the boundary conditions of when justice preferences shift between punitive and non-punitive responses, while also providing a mechanism for why punishment is not systematically endorsed across all social contexts.

Our data suggest that there are only some situations where punishment is preferred over non-punitive options. Even if punishment is free and easy to enact, people are less motivated to punish if they are already amply compensated. Moreover, so long as compensation is available, decisions (but not judgments) to punish are largely not proportional to the degree of infraction. While this stands in stark contrast to the large amount of evidence illustrating that people have a strong desire to punish^11,38^, it is possible that previous work may have inflated punitive preferences because participants were not able to select non-punitive options for restoring justice. Our results suggest that when the transgression is relatively minor, punishment may not be preferred nor needed when non-punitive alternatives are available to rectify the fairness violation. However, future research—such as field experiments—can explore whether preferences for compensation extend to severe moral transgressions that are not hypothetical in nature.

Dovetailing with evolutionary accounts about reputation^47,48^, we find that how one decides to restore justice can signal information about one’s moral (or immoral) character. Individuals who endorse prosocial responses to moral infractions are perceived as more valued social partners than those who express antisocial preferences for restoring justice. Indeed, endorsing prosocial responses may have an effect on positive emotions and overall wellbeing^49^. However, the reputational impact of these decisions is modulated by the perspective of the deciding agent. When people decide to punish on behalf of another individual, they are more desired as social partners and are perceived as having better moral character than individuals who punish as a victim. This likely because third-party observers are adhering to and upholding fundamental moral norms, in this case punishing fairness violations^23,26,27^. Future studies leveraging manipulations can more directly examine whether this relationship is causal in nature.

In contrast, failing to punish or taking no action as a third-party (e.g., accepting the unfair offer without any attempt at restoring justice) may be interpreted as condoning the transgression, such that the individual is neither aware nor willing to uphold moral norms. In these cases, failing to act is perceived as a signal of a questionable moral character. In a similar vein, not wanting to engage with victims who punished perpetrators suggests that such antisocial behaviors are perceived in a negative moral light. For instance, it is possible that victims who punish are perceived as trying to get ‘payback’ or acting on vindictive motives. Together, these results demonstrate that the reputational value associated with punishment is malleable, and critically changes depending on the perspective of the individual enacting justice.

Even the reputational signal of third-party punishment, however, seems to hinge on the social context. In situations where different moral values such as altruism and generosity are prioritized, we find that third-party members who punish are preferred *less* compared to those who responded in a prosocial manner. One possibility for why third-party punishers are not selected as social partners in a Dictator Game is because decisions to punish provide little information about whether a person values altruism. For example, there does not seem to be a relationship between real world altruistic generosity and decisions to punish^50^, and recent work illustrates that punishing can either be construed as altruistically enforcing a social norm or spitefully responding to a perpetrator^45^. Thus, in these cases, punishment may be sending an ambiguous signal about an individual’s moral character. In contrast, endorsement of other choices in the Justice Game likely sends more informative signals about a person’s altruistic tendencies and what they care to uphold. For example, choosing compensation or equity could signal that the person clearly, and unambiguously, cares about prosocial values, including altruism or generosity. These results provide compelling evidence that third-party punishment is only perceived as a positive moral signal in limited social contexts.

Decades of research have examined people’s preference for punishment and its suggested role in enforcing normative behavior. And yet, our data suggests there is a strongly held preference for non-punitive justice restoration. These findings mirror evidence from outside the lab. A 2016 National Survey of Victims’ Views found that victims overwhelmingly prefer that the United States criminal justice system focus more on rehabilitation than punishment^51^. Together, these findings have implications for criminal justice systems; if we want justice restoration to reflect the values and preferences of its citizens, then non-punitive alternatives for alleviating wrongdoing should be promoted more than they are now.

## Supplementary information

Supplement
