# Supplementary material for: Why we don’t always punish: Preferences for non-punitive responses to moral violations
Source: Sci Rep. 2019 Sep 13;9:13219. doi: 10.1038/s41598-019-49680-2 (PMC6744396; doi:10.1038/s41598-019-49680-2)
Supplement: Supplementary file 1 — Supplement [file 41598_2019_49680_MOESM1_ESM.docx]

Supplementary information

*for*

**Why we don’t always punish: Preferences for non-punitive responses to moral violations**

Joseph Heffner and Oriel FeldmanHall

Table of Contents

[1 Experiment 1 2](#_Toc16069814)

[1.1 Methods 2](#_Toc16069815)

[**Experimental design overview** 2](#_Toc16069816)

[**Task instructions** 2](#_Toc16069817)

[1.2 Results 3](#_Toc16069818)

[2 Experiment 2 9](#_Toc16069819)

[2.1 Methods 9](#_Toc16069820)

[**Experimental design overview** 9](#_Toc16069821)

[**Task instructions** 9](#_Toc16069822)

[2.2 Results 10](#_Toc16069823)

[3 Experiment 3 12](#_Toc16069824)

[3.1 Methods 12](#_Toc16069825)

[**Experimental design overview** 12](#_Toc16069826)

[**Participants** 12](#_Toc16069827)

[**Crime Vignettes** 12](#_Toc16069828)

[**Task Instructions** 16](#_Toc16069829)

[3.2 Results 17](#_Toc16069830)

[4 Experiment 4 18](#_Toc16069831)

[4.1 Methods 18](#_Toc16069832)

[**Experimental design overview** 18](#_Toc16069833)

[**Task instructions** 18](#_Toc16069834)

[4.2 Results 20](#_Toc16069835)

[5 Experiment 5 23](#_Toc16069836)

[5.1 Methods 23](#_Toc16069837)

[**Experimental design overview** 23](#_Toc16069838)

[**Hypothetical Vignette** 23](#_Toc16069839)

[**Task Instructions** 24](#_Toc16069840)

[References 25](#_Toc16069841)

# 1 Experiment 1

## 1.1 Methods

**Experimental design overview**. In Experiment 1, we employ a modified Justice Game (JG) which juxtaposes compensation and punishment as possible methods for justice restoration. To isolate the respective effects of compensation and punishment there are two trial types in the JG: partial compensation and partial punishment trials (detailed in the manuscript).

**Task instructions**. Participants were given instructions for both the victim and third-party conditions. Participants were also walked through an example trial and took comprehension questions to ensure they understood Player A’s offers.

*Instructions for Experiment 1*

*The purpose of this task is to study how people make decisions. You will be making real decisions that affect the monetary outcomes of YOURSELF and OTHERS. You will be playing multiple rounds of a game. Each round will be one of two scenarios. You will be informed of which scenario you are playing at the start of each round.*

*Scenario 1: You are Player B*

*Scenario 2: You are Player C*

*In both scenarios, Player A has been allotted $1.00. Each Player A (you will play with a different person on each round) has already decided how much of their $1 to share with Player B. For example, Player A can decide to:*

*1. keep $.90 and give $.10*

*2. keep $.80 and give $.20*

*3. keep $.70 and give $.30*

*4. keep $.60 and give $.40*

*5. keep $.50 and give $.50*

*After observing Player A make a split, you will be asked to determine the monetary outcome of both Player A and Player B. You decide how much money Player A and Player B get based on two available options. These options can be some combination of the following:*

*1. Decrease Player A’s money (thereby punishing them for an unfair offer)*

*2. Increase Player B’s money (thereby compensating them for receiving an unfair offer)*

*3. Keep both Player’s money the same*

*Ultimately, you will decide how much money Player A and B actually receive.*

***IMPORTANT: 

You will be playing multiple rounds of this game. Sometimes as Player B and sometimes as Player C.***

***In Scenario 1, you are making the choice for your own monetary outcome. In other words, you will have a personal stake in the outcomes, and you will have the chance to make additional money depending on your choices.

In Scenario 2, you are making the choice on behalf of a 3rd person, Player B.  That is, you will not yourself be invested in the decision when you are deciding as Player C, but you will make choices that will affect the monetary outcomes of another Player B. When you are making decisions as Player C, you will not make an additional bonus but Players A and B could make additional money depending on your choices.***

## 1.2 Results

*Effect of fairness violations on decisions to punish as the victim*. Using a logistic mixed-effects model, we tested our hypothesis that increasing compensation for the victim (Player B) would result in less punitive behaviors toward the perpetrator (Player A) across all possible fairness violations.

Across all levels of unfairness, victims punish less as compensation increases (Fig. S1 and Table S1). Once participants are fully compensated (i.e., maximal compensation), punishment is no longer the most preferred decision across all unfairness levels.

***
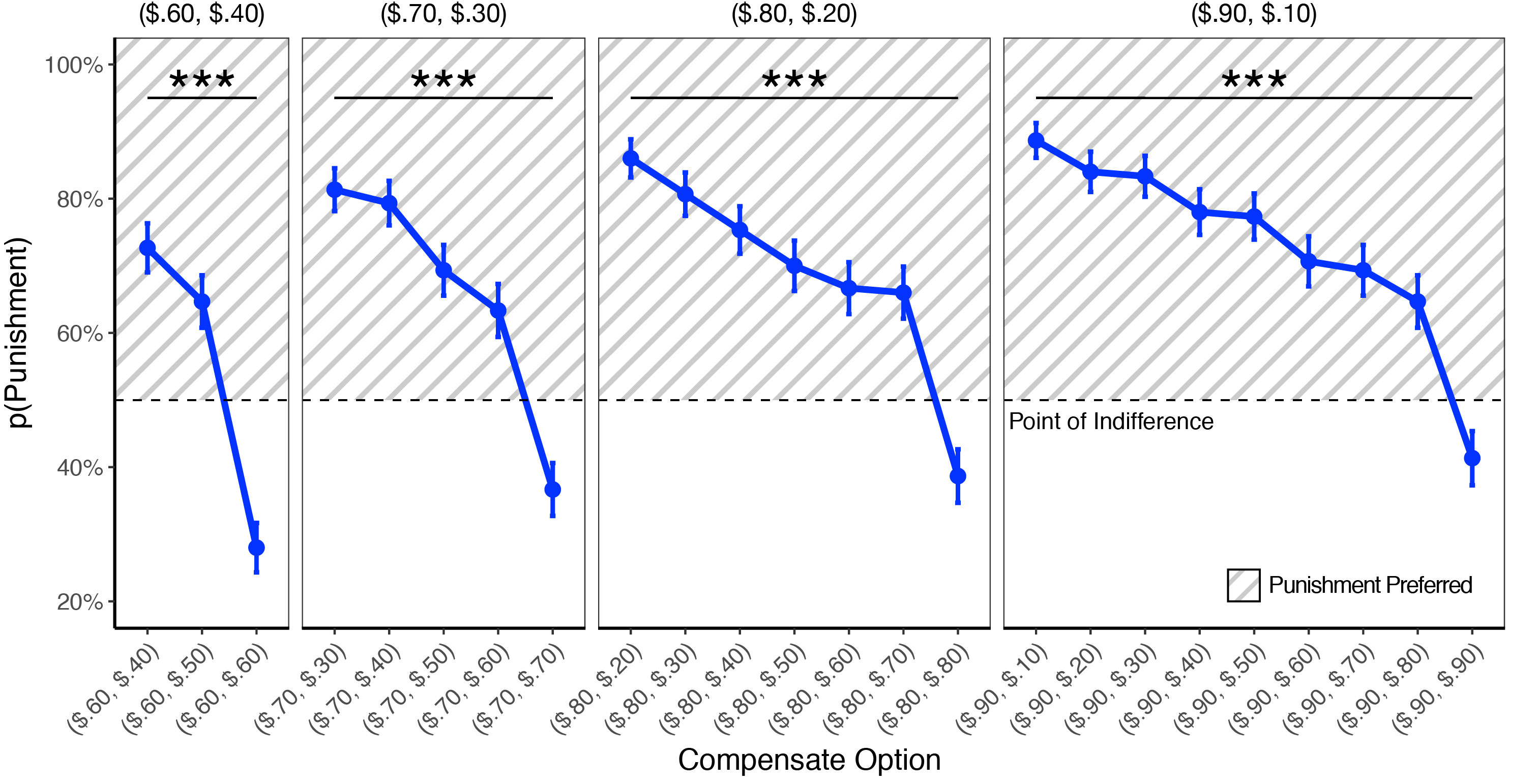
***

***Figure S1. Preference for punishment for all unfairness levels when responding as the victim.*** *We computed selection of maximal punishment juxtaposed across all partial compensation options for each level of unfairness. For example, the second data point in a relatively fair offer ($.60, $.40) shows a trial where participants chose between maximal punishment ($.40, $.60) and a form of partial compensation ($.60, $.50). Each data point represents a $.10 increase in compensation from minimal to maximal (changes depending on the unfairness level). The point of indifference indicates a 50% chance of choosing punishment and the shaded area visualizes the area where punishment is the preferred option. Error bars are ±1 SEM.*

**Table S1. Compensation and unfairness influence decisions to punish as the victim**

| ${Punishment}_{i,t}= \beta_{0}+ \beta_{1}{Compensation}_{i, t} \times\beta_{2}{Unfairness}_{i, t}+ \varepsilon$ | | | | |
| --- | --- | --- | --- | --- |
| Dependent variable | Estimate (SE) | t | p |  |
| Decisions to Punish |  |  |  |  |
| Intercept | 3.67 (0.51) | 7.12 | <.001*** |  |
| Compensation | -1.52 (0.17) | -8.73 | <.001*** |  |
| Unfairness | 1.61 (0.21) | 7.78 | <.001*** |  |
| Compensation $\times$ Unfairness | 0.05 (0.04) | 1.21 | .22 |  |
| *Note.* Punishment ~ Compensation $\times$ Unfairness, where Punishment is coded as (1) if chosen, and elsewise 0. Compensation and Unfairness are indexed by participant and trial. Compensation is a continuous variable, ranging from minimal (e.g., Player B receives $.10) to maximal (e.g., Player B receives $.90), depending on unfairness of the split, and has been mean-centered. Unfairness was mean centered before being entered into the regression. The model includes a random intercept and a random slope for compensation and unfairness per subject.  ***p <.001 | | | | |

*Effect of role (victim versus third-party) on decisions to punish***.** Participants also made decisions as a third-party (within-subject design). By designing a task where subjects had to make decisions both as a victim and third-party, we can measure whether preference for punitive and non-punitive options changes depending on the perspective of the deciding agent. Results reveal an interaction between role and compensation such that as monetary compensation increases, the effect of role diminishes (Table S2).


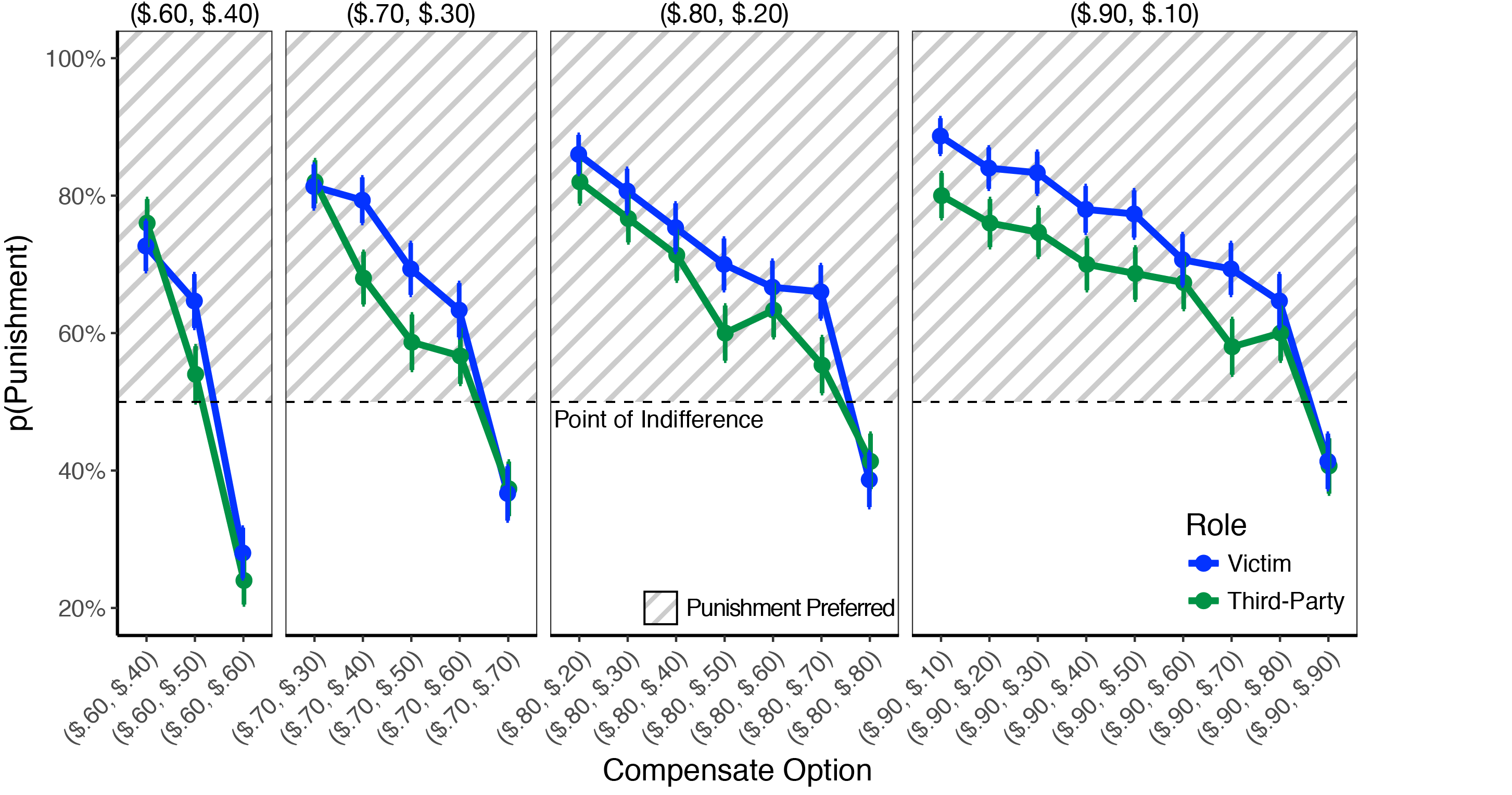


***Figure S2. Preference for punishment for all unfairness levels by role (victim versus third-party).*** *Preference for punishment in response to unfair offers ranging from a mostly fair offer ($.60, $.40) to a highly unfair offer ($.90, $.10), deciding as the victim or third-party. Error bars are ±1 SEM.*

**Table S2. Effect of role (victim or third-party) on decisions to punish across unfairness levels**

| ${Punishment}_{i,t}= \beta_{0}+\beta_{1}{Role}_{i, t} \times\beta_{2}{Compensation}_{i, t}\times\beta_{3}{Unfairness}_{i, t}+ \varepsilon$ | | | |
| --- | --- | --- | --- |
| Dependent variable | Estimate (SE) | t | p |
| Decisions to Punish |  |  |  |
| Intercept | 3.42 (0.45) | 7.68 | <.001*** |
| Role | -0.64 (0.16) | -3.93 | <.001*** |
| Compensation | -1.34 (0.14) | -9.78 | <.001*** |
| Unfairness | 1.49 (0.17) | 8.52 | <.001*** |
| Role $\times$ Compensation | 0.09 (0.06) | 1.67 | .09 |
| Role $\times$ Unfairness | -0.21 (0.08) | -2.66 | .008** |
| Compensation $\times$ Unfairness | 0.13 (0.04) | 3.38 | <.001*** |
| Role $\times$ Compensation $\times$ Unfairness | 0.06 (0.05) | 1.12 | .26 |
| *Note.* Punishment ~ Role $\times$ Compensation $\times$ Unfairness, where Punishment is coded as (1) if chosen, and elsewise 0. Role, Compensation and Unfairness are indexed by participant and trial. Role is coded as (0) for Victim and (1) for Third-Party. Compensation is a continuous variable, ranging from minimal (e.g., Player B receives $.10) to maximal (e.g., Player B receives $.90), depending on unfairness of the split, and has been mean-centered. Unfairness was mean centered before being entered into the regression. The model includes a random intercept and a random slope for compensation, unfairness, and role per subject.  *p < .05 **p < .01 ***p <.001. | | | |

*Effect of fairness violations on decisions to punish when responding as the victim*. Using a logistic mixed effects model, we tested our hypothesis that when the victim’s needs are met (i.e., when maximal compensation is available), no amount of punishment will be valued. For all levels of fairness infraction, participants preferred the non-punitive, compensatory response over partial punishment options (Fig. S3; Table S3).


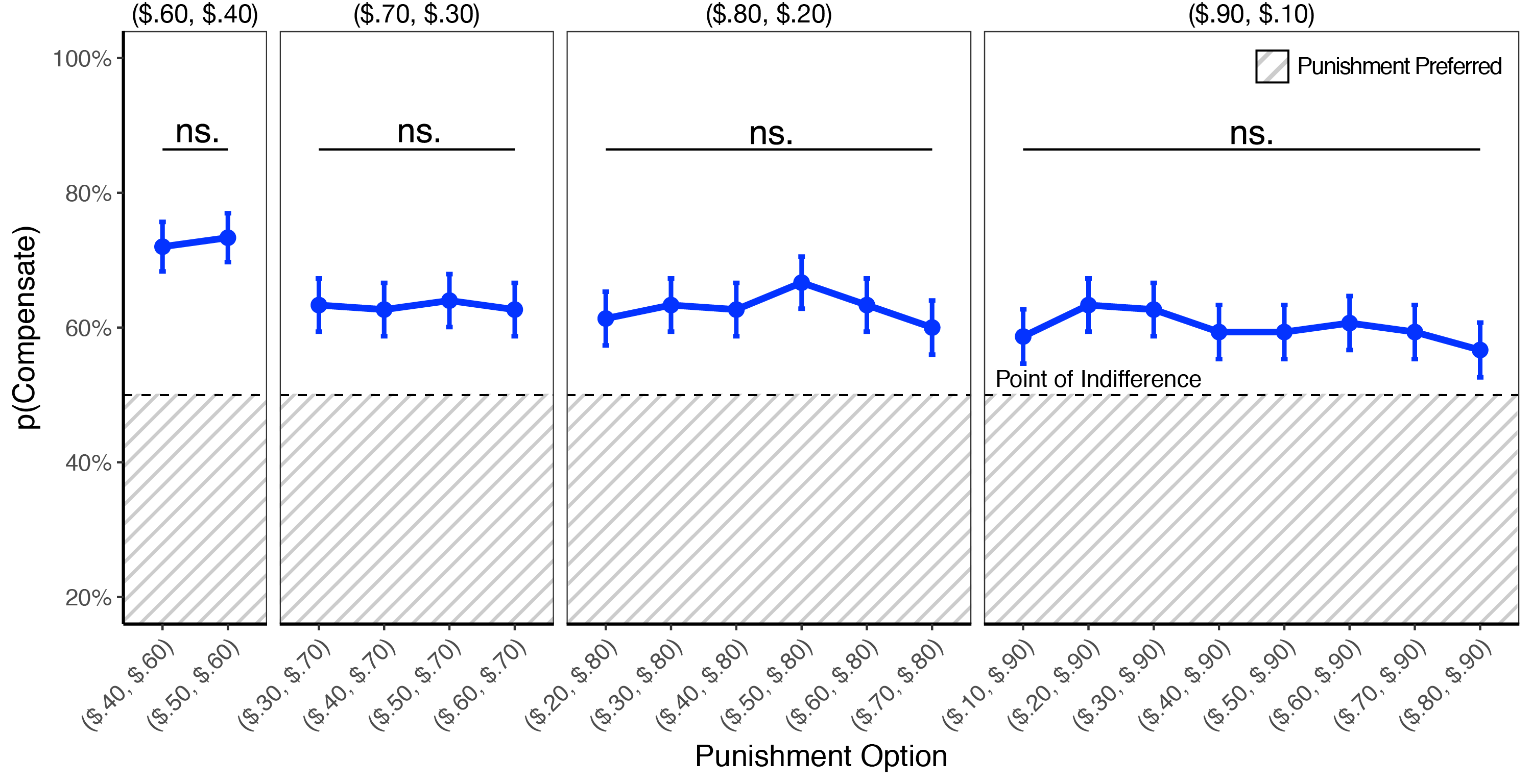


***Figure S3. Preference for compensation for all unfairness levels when responding as the victim.*** *We computed selection of maximal compensation juxtaposed across all partial punishment options for each level of unfairness. For example, the first data point in a relatively fair offer ($.60, $.40) shows a trial where participants chose between maximal compensation ($.60, $.60) and maximal punishment ($.40, $.60). Each data point represents a $.10 decrease* *in punishment from maximal to minimal, which changes depending on the unfairness level. The point of indifference indicates a 50% chance of choosing punishment and the shaded area visualizes when punishment is the preferred option. Error bars are ±1 SEM.*

**Table S3. Punishment and Unfairness do not Influence Decisions to Compensate as the Victim**

| ${Compensate}_{i,t}= \beta_{0}+ \beta_{1}{Punishment}_{i, t} \times\beta_{2}{Unfairness}_{i, t}+ \varepsilon$ | | | |
| --- | --- | --- | --- |
| Dependent variable | Estimate (SE) | t | p |
| Decisions to Punish |  |  |  |
| Intercept | 1.85 (0.56) | 3.29 | <.001*** |
| Punishment | -0.03 (0.05) | -0.57 | .57 |
| Unfairness | 0.03 (0.21) | 0.15 | .88 |
| Punishment $\times$ Unfairness | -0.05 (0.06) | -0.93 | .35 |
| *Note.* Compensate ~ Punishment $\times$ Unfairness, where Compensate is coded as (1) if chosen, and elsewise 0. Punishment and Unfairness are indexed by participant and trial. Punishment is a continuous variable ranging from minimal (e.g., Player A receives $.80) to maximal (e.g., Player A receives $.10) depending on unfairness of the split, and has been mean-centered. Unfairness was mean centered before being entered into the regression. The model includes a random intercept and a random slope for unfairness per subject.  **p < .01 | | | |

*Effect of role (victim versus third-party) on decisions to compensate*

When we include role (e.g., deciding as the victim or third party) in our logistic regression, we find no interaction between the degree of punishment and whether participants decided as the victim or a third-party (Fig. S4 and Table S4).


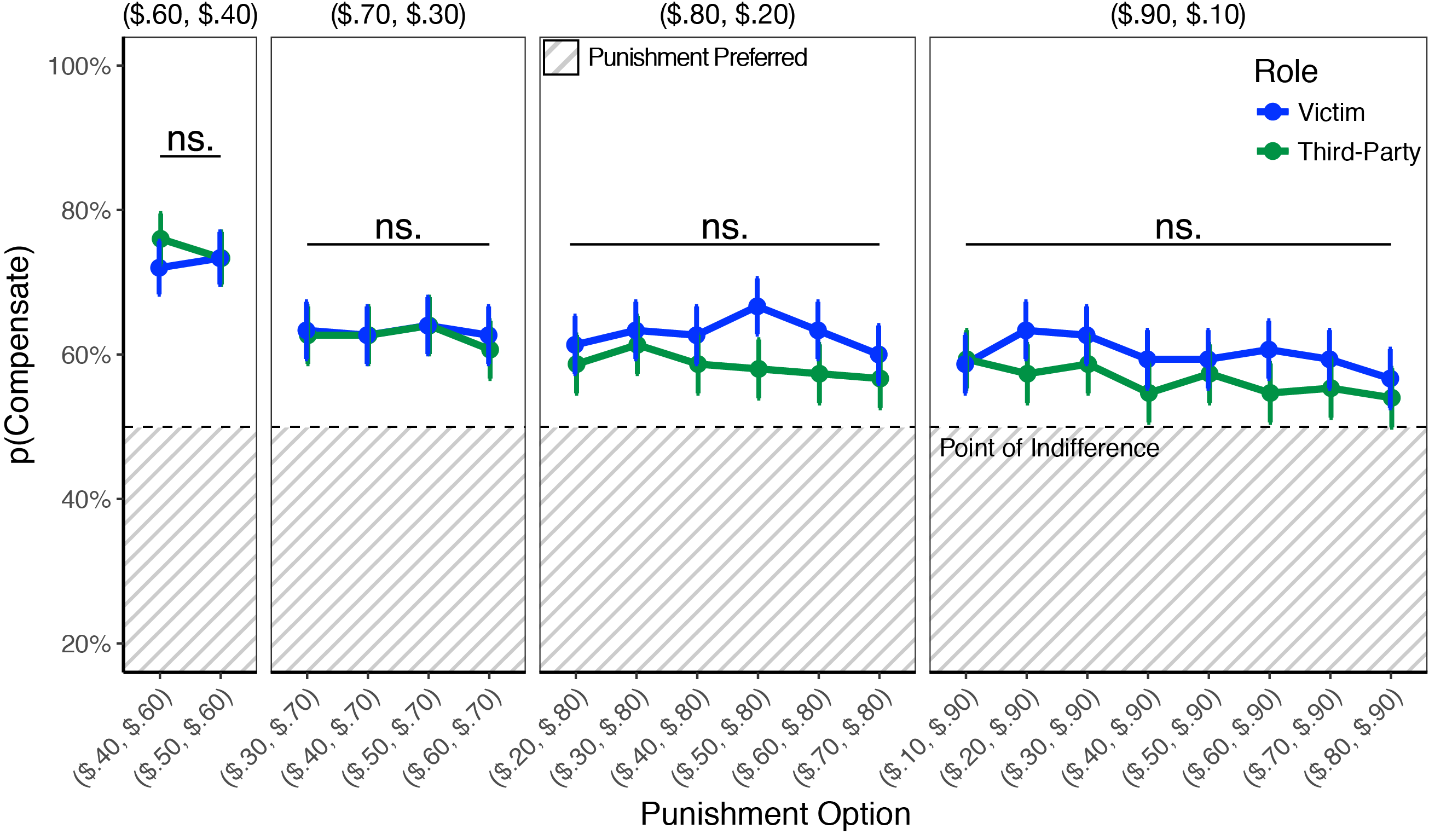


***Figure S4. Preference for compensation for all unfairness levels indexed by Role.*** *Preference for compensation in response to unfair offers ranging from a relatively fair offer ($.60, $.40) to a highly unfair offer ($.90, $.10). Error bars are ±1 SE.*

**Table S4. Effect of role (victim or third-party) on decisions to compensate**

| ${Compensate}_{i,t}= \beta_{0}+\beta_{1}{Role}_{i, t} \times\beta_{2}{Punishment}_{i, t}\times\beta_{3}{Unfairness}_{i, t}+ \varepsilon$ | | | |
| --- | --- | --- | --- |
| Dependent variable | Estimate (SE) | t | p |
| Decisions to Punish |  |  |  |
| Intercept | 1.57 (0.46) | 3.42 | <.001*** |
| Role | -0.41 (0.12) | -3.36 | <.001*** |
| Punishment | -0.02 (0.05) | -0.37 | .71 |
| Unfairness | -0.22 (0.15) | -1.50 | .13 |
| Role $\times$ Punishment | -0.06 (0.07) | -0.95 | .34 |
| Role $\times$ Unfairness | -0.28 (0.09) | -2.98 | .003** |
| Punishment $\times$ Unfairness | -0.04 (0.05) | -0.82 | .41 |
| Role $\times$ Punishment $\times$ Unfairness | .04 (0.07) | 0.59 | .56 |
| *Note.* Compensate ~ Role $\times$ Punishment $\times$ Unfairness, where Compensate is coded as (1) if chosen, and elsewise 0. Role, Punishment, and Unfairness are indexed by participant and trial. Role is coded as (0) for Victim and (1) for Third-Party. Punishment is a continuous variable ranging from minimal (e.g., Player A receives $.80) to maximal (e.g., Player A receives $.10) depending on unfairness of the split, and has been mean-centered. Unfairness was mean centered before being entered into the regression. The model includes a random intercept and a random slope for punishment, unfairness, role per subject.  **p < .01 ***p <.001. | | | |

# 2 Experiment 2

## 2.1 Methods

**Experimental design overview**. In Experiment 2, participants again played the Justice Game but used a sliding visual analogue scale (VAS) to determine the final monetary outcomes of both Player A and Player B. The VAS’s indicator bar always started at $0 for both Player A and Player B and participants selected a non-zero monetary payout in $.10 increments for each player (minimum is $.10). Participants were explicitly told that the total monetary amounts of both players do not have to add up to $1.

**Task instructions**. Participants were given instructions for both the victim and third-party conditions. Participants were also walked through an example trial and took comprehension questions to ensure they understood Player A’s offers.

*Instructions for Experiment 2*

*The purpose of this task is to study how people make decisions. You will be making real decisions that affect the monetary outcomes of YOURSELF and OTHERS. You will be playing multiple rounds of a game. Each round will be one of two scenarios. You will be informed of which scenario you are playing at the start of each round.*

*Scenario 1: You are Player B*

*Scenario 2: You are Player C*

*In both scenarios, Player A has been allotted $1.00. Each Player A (you will play with a different person on each round) has already decided how much of their $1 to share with Player B. For example, Player A can decide to:*

*1. keep $.90 and give $.10*

*2. keep $.80 and give $.20*

*3. keep $.70 and give $.30*

*4. keep $.60 and give $.40*

*5. keep $.50 and give $.50*

*Once Player A has made a split, you can decide the monetary outcomes of both Player A and Player B using a sliding scale. You can keep the monetary outcomes the same, decrease, or increase Player A and/or Player B without these amounts being contingent upon each other. This is to say that the total amount does NOT have to add up to $1, you can choose any amount of money for Player A and Player B using the sliding scale.*

*Once you make your selection and go to the next page, your decision will be recorded and the next trial will appear, so please be certain of your choice before advancing.*

*You will be playing multiple rounds of this game. Sometimes as Player B and sometimes as Player C.*

*In Scenario 1, you are making the choice for your own monetary outcome. In other words, you will have a personal stake in the outcomes, and you will have the chance to make additional money depending on your choices.*

*In Scenario 2, you are making the choice on behalf of a 3rd person, Player B. That is, you will not yourself be invested in the decision when you are deciding as Player C, but you will make choices that will effect the monetary outcomes of another Player B. When you are making decisions as Player C, you will not make an additional bonus but Players A and B could make additional money depending on your choices.*

## 2.2 Results

We found no evidence that decisions to punish shifted depending on whether the participant was deciding as a victim or third-party. Across both roles, preferences for compensation to Player B increases as the fairness violation increases, however the amount of money redistributed to Player A remains consistent at roughly $.50, regardless of how unfair the offer was (Fig. S5 and Table S5).


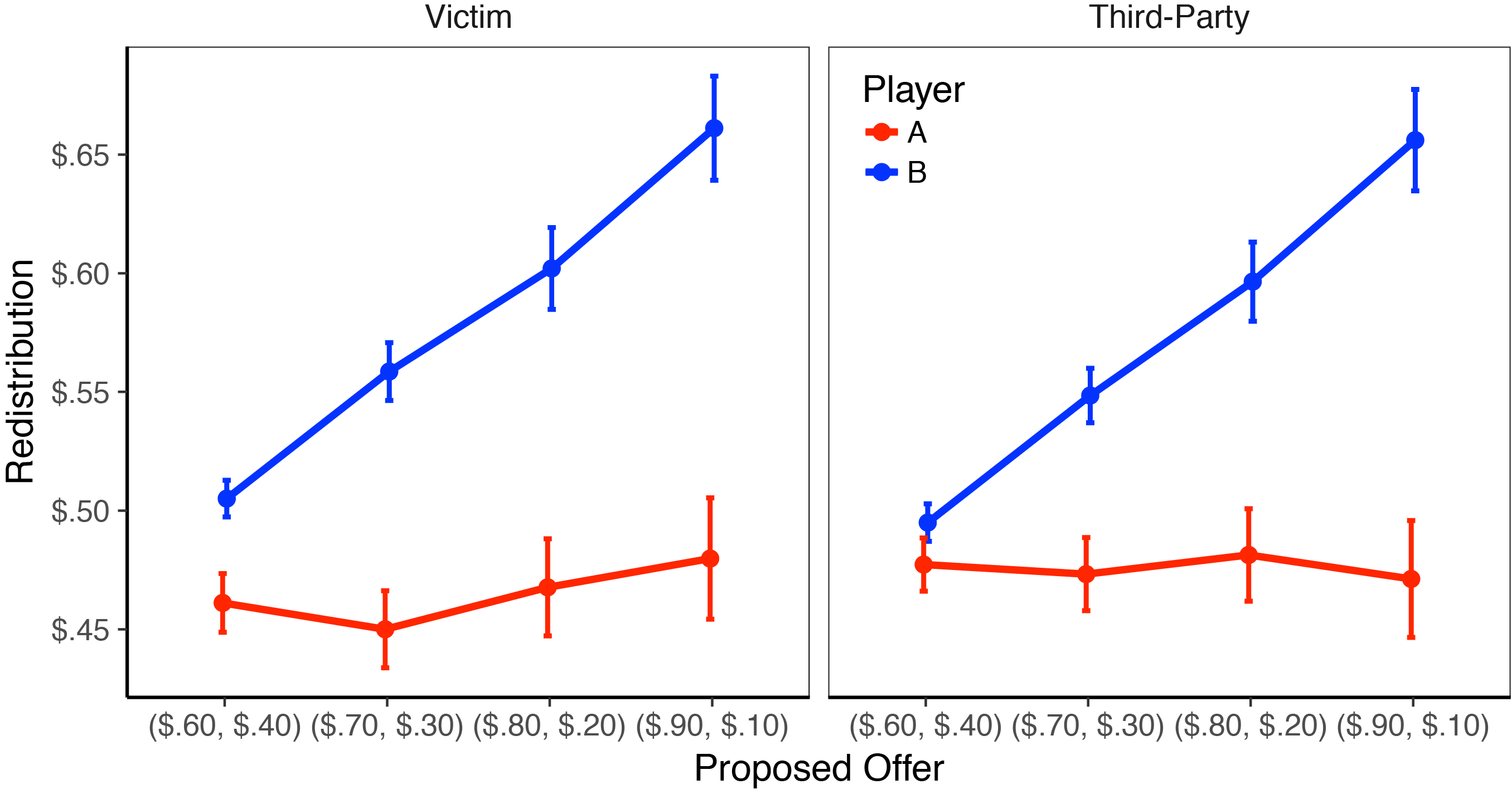


***Figure S5. Unconstrained Redistributions across Role****. Average monetary redistribution is plotted for Player A (in red) and Player B (in blue) as a function of the proposed offer and by role (victim or third-party). Error bars are ±1 SEM.*

**Table S5. Participants prefer redistributions which compensate Player B instead of punish Player A across roles**

| ${Redistribution}_{i,t}= \beta_{0}+ \beta_{1}{Role}_{i, t}\times\beta_{2}{Player}_{i, t}\times\beta_{3}{Unfairness}_{i, t}+ \varepsilon$ | | | |  |
| --- | --- | --- | --- | --- |
| Dependent variable | Estimate (SE) | t | p | |
| Redistribution |  |  |  | |
| Intercept | 47.84 (1.63) | 29.30 | <.001*** | |
| Role | -1.12 (0.69) | -1.63 | .10 | |
| Player | 9.49 (2.45) | 3.87 | <.001*** | |
| Unfairness | -0.005 (.44) | -0.01 | .99 | |
| Role × Player | 2.04 (.97) | 2.09 | .04* | |
| Role × Unfairness | 0.80 (.62) | 1.30 | .19 | |
| Player × Unfairness | 5.27 (0.62) | 8.55 | <.001*** | |
| Role × Player × Unfairness | -0.87 (.87) | -0.99 | .32 | |
| *Note.* Redistribution ~ Player × Unfairness × Role, where Redistribution is a continuous variable ranging from 10 cents to 90 cents. Player, Unfairness Level, & Role are indexed by participant and trial. Player A serves as the reference (0) for Player. Unfairness was mean centered before being entered into the regression. Role is coded as (1) for Victim and (0) for Third-Party. The model includes a random intercept and a random slope for Player per subject.  * p < .05. ** p < .01. *** p <.001. | | | |  |
|  | | | |  |

# 3 Experiment 3

## 3.1 Methods

**Experimental design overview**. In Experiment 3, participants were presented with short vignettes of a variety of crimes and were asked to judge the amount of compensation for the victim and punishment for the perpetrator. Using a between-subjects design, participants were either asked to make these judgments as the hypothetical victim of the crime or a third-party member. The order of the crimes was fully randomized.

**Participants**. In our sample of 271 participants, 134 completed the victim condition, while 137 (89 women; age = 31.9 years, SD ± 8.7) completed the third-party condition.

**Crime Vignettes**. We pulled crime examples from the “Uniform Crime Reporting Handbook” published by the Federal Bureau of Investigation^1^, a publication which establishes uniform definitions for crimes based on a hierarchical classification procedure. The victim crime vignettes (Table S6) and third-party crime vignettes (Table S7) are listed below with their label and descriptions.

**Table S6. Victim Crime Vignettes**

| Label | Description |
| --- | --- |
| 1. Murder & nonnegligent manslaughter | “You and your spouse had an argument. Afterward, your spouse shot you and you nearly died.” |
| 2. Manslaughter by negligence | “A target shooter was practicing in a wooded area near your house. One shot missed the target and hit you. You nearly died.” |
| 3. Forcible rape | “As you were leaving work, you were attacked in the parking lot by an unidentified male and forcibly raped.” |
| 4. Attempts to commit forcible rape | “A man attacked you on the street, attempting to rape you. A pedestrian frightened the man away before he could complete the attack.” |
| 5. Robbery with firearm | “A man with a gun appeared in a bar and ordered you to hand over your cash. After obtaining the money, the man left.” |
| 6. Robbery with knife or cutting instrument | “You were walking down the street when an assailant grabbed you and held a knife to your throat. The assailant removed your wallet from your pocket and ran.” |
| 7. Robbery with other dangerous weapon | “A man accosted you in an alley near a bar. He beat you severely with a club and took your wallet.” |
| 8. Robbery with strong-arm (hands, fists, feet, etc.) | “During a bag snatching, a thief shoved you to the ground and took your bag that had your wallet in it.” |
| 9. Aggravated assault with firearm | “While you were picking up some food, an individual fired at you, wounding but not killing you.” |
| 10. Aggravated assault with knife or cutting instrument | “During a baseball game, a heated argument erupted between you and a man. The man stabbed you with a knife.” |
| 11. Aggravated assault with other dangerous weapon | “During an argument, a man picked up a tire iron and hit you. You had a concussion from a blow to the head.” |
| 12. Aggravated assault with strong-arm (hands, fists, feet, etc.) | “Your spouse came home drunk. During an argument with you, they slapped you with an open hand and broke your jaw.” |
| 13. Burglary with forcible entry | “A burglar used a lock pick to enter your house and stole numerous items from you.” |
| 14. Burglary with unlawful entry (no force) | “During the night, a man stole your car out of your unlocked, but closed, private garage.” |
| 15. Larceny-theft | “While standing in a crowd watching a parade, you were jostled by someone who stole your wallet.” |
| 16. Motor vehicle theft (auto) | “You stopped at a mailbox and left your car running while you got out to mail a letter. A man jumped into the vehicle and drove away.” |
| 17. Arson (structural) | “As the result of a fire, your house was destroyed. Investigation revealed an arsonist had ignited the fire.” |
| 18. Arson (mobile) | “A man threw a firebomb at your parked vehicle; the device hit your car and set it on fire.” |
| 19. Other assaults | “One male student threatens to beat you up unless you hand over your lunch money.” |
| 20. Forgery and counterfeiting | “A man steals your checkbook and writes a check for himself by forging your signature.” |
| 21. Fraud | “A man constructed a device which allowed him to take your money from an ATM.” |
| 22. Embezzlement | “A friend of yours steals money from your bank account over a period of many months.” |
| 23. Stolen property: buying, receiving, possessing | “A man buys your stolen television from a stranger.” |
| 24. Vandalism | “A man walks into a parking lot and slashes the tires of your automobile.” |
| 25. Sex offenses | “A man exposes his genitals to you while you are sitting on a park bench.” |
| 26. Offenses against the family and children | “Your ex-spouse, tired of paying alimony, decides to leave town to avoid you.” |
| 27. Disorderly conduct | “A drunken man stumbles outside of a bar and yells out vulgarities at you.” |
| 28. All other offenses (everything but traffic) | “An ex-lover blackmails you by threatening to send naked photos of you to your friends unless you pay them a large sum of money.” |
| 29. All other offenses (everything but traffic) | “A hotel doorman threatens to tell your spouse about your numerous affairs at the hotel unless you pay him a large sum of money.” |

**Table S7. Third-Party Crime Vignettes**

| Label | Description |
| --- | --- |
| 1. Murder & nonnegligent manslaughter | “A husband and wife had an argument. Afterward, the wife shot the husband and killed him.” |
| 2. Manslaughter by negligence | “A target shooter was practicing in a wooded area near some houses. One shot missed the target and killed a resident.” |
| 3. Forcible rape | “As a woman was leaving work, she was attacked in the parking lot by an unidentified male and forcibly raped.” |
| 4. Attempts to commit forcible rape | “A man attacked a woman on the street, attempting to rape her. A pedestrian frightened the man away before he could complete the attack.” |
| 5. Robbery with firearm | “A man with a gun appeared in a bar and ordered the bar owner to hand over your cash. After obtaining the money, the man left.” |
| 6. Robbery with knife or cutting instrument | “A man was walking down the street when an assailant grabbed him and held a knife to his throat. The assailant removed the victim’s wallet from his pocket and ran.” |
| 7. Robbery with other dangerous weapon | “A man accosted another man in an alley near a bar. He beat the victim severely with a club and took his wallet.” |
| 8. Robbery with strong-arm (hands, fists, feet, etc.) | “During a purse-snatching, a thief shoved a woman to the ground and took her purse.” |
| 9. Aggravated assault with firearm | “While an officer was attempting to serve a warrant, the individual fired on the officer, wounding but not killing her.” |
| 10. Aggravated assault with knife or cutting instrument | “During a baseball game, a heated argument erupted and one man stabbed another with a knife.” |
| 11. Aggravated assault with other dangerous weapon | “During an argument, a man picked up a tire iron and hit his female neighbor. The woman had a concussion from a blow to the head.” |
| 12. Aggravated assault with strong-arm (hands, fists, feet, etc.) | “A man came home drunk. During an argument with his wife, he slapped her with an open hand and broke her jaw.” |
| 13. Burglary with forcible entry | “A burglar used a lock pick to enter a house and stole numerous items from the owner.” |
| 14. Burglary with unlawful entry (no force) | “During the night, a man stole a car out of an unlocked, but closed, private garage.” |
| 15. Larceny-theft (*larceny*) | “While standing in a crowd watching a parade, a man was jostled by someone who stole his wallet.” |
| 16. Motor vehicle theft (auto) | “A woman stopped at a mailbox and left her minivan running while she got out to mail a letter. A man jumped into the vehicle and drove away.” |
| 17. Arson (structural) | “As the result of a fire, someone’s house was destroyed. Investigation revealed an arsonist had ignited the fire.” |
| 18. Arson (mobile) | “A man threw a firebomb at a parked vehicle; the device hit the car and set it on fire.” |
| 19. Other assaults | “One male student threatens to beat up another student unless he hand over his lunch money.” |
| 20. Forgery and counterfeiting | “A man steals a woman’s checkbook and writes a check for himself by forging her signature.” |
| 21. Fraud | “A man constructed a device which allowed him to take a stranger’s money from an ATM.” |
| 22. Embezzlement | “A caretaker of an elderly man steals money from his bank account over a period of many months.” |
| 23. Stolen property: buying, receiving, possessing | “A man buys a television from a stranger who told him that he stole it from his employer.” |
| 24. Vandalism | “A man walks into a parking lot and slashes the tires of an automobile.” |
| 25. Sex offenses | “A man exposes his genitals to a woman sitting on a park bench.” |
| 26. Offenses against the family and children | “A man, tired of paying alimony, decides to leave town to avoid his ex-wife.” |
| 27. Disorderly conduct | “A drunken man stumbles outside of a bar and yells out vulgarities at a passing woman.” |
| 28. All other offenses (everything but traffic) | “An ex-boyfriend blackmails an ex-girlfriend by threatening to send naked photos of her to their friends unless she pays him a large sum of money.” |
| 29. All other offenses (everything but traffic) | “A hotel doorman threatens to tell a man’s wife about his numerous affairs at the hotel unless he pays him a large sum of money.” |

**Task Instructions**. Participants were only given one set of instructions for either victim or third-party conditions. Participants were walked through an example crime and took comprehension questions to ensure they could identify aspects of the crime.

*Instructions for Experiment 3 Victim condition (third-party condition changes in parentheses)*

*The purpose of this task to is understand your perceptions of crime. You will read different descriptions of crimes and make judgments about them. In all of the descriptions, you will be the victim of the crime. After reading the description, you will make 3 judgments about the crime:*

*1. How much should you (the victim)* *be compensated for the crime?*

*2. How much should the perpetrator be punished for the crime?*

*3. Please rate the moral severity of the crime.*

*Please note that there are NO right or wrong answers for these questions. We are simply interested in your judgements.*

*Definitions:*

***Compensation*** *is defined as "something, typically money, awarded to someone as a recompense for loss, injury, or suffering."*

***Punishment*** *is defined as "the infliction or imposition of a penalty as retribution for an offense."*

***Moral severity*** *is defined as "the degree to which a moral violation is wrong."*

*PLEASE TAKE YOUR TIME TO READ THE DESCRIPTIONS THOROUGHLY.*

## 3.2 Results

We conducted a linear mixed-effects regression predicting amount of punishment/compensation as a function of judgment type (punishment or compensation) and degree of moral severity for third parties. Results revealed that third-parties were influenced by moral severity in a similar way as victims: third-party observers gave harsher punishments to the perpetrator and more generous compensation to the victim as the perceived moral severity of the crime increased (Fig. S6 and Table S8).


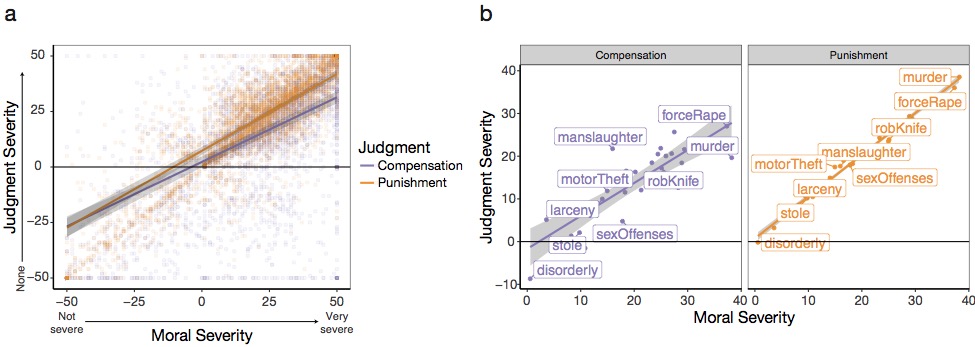


***Figure S6. Crime results for third-parties (a) Results****. Judgment severity is plotted for compensation (in purple) and punishment (in orange) as a function of perceived moral severity for third-party observers. Plot lines reflect parameter fits based on trial-wise mixed-effects regression from Table S7. Shaded error bars reflect 95-percent confidence intervals.* ***(b) Results grouped by crime.*** *For each crime vignette, we averaged participants’ judgments of moral severity, compensation, and punishment. Average amount of compensation and punishment allotted is plotted as a function of the perceived moral severity of each crime. A subset of the 29 crimes are labeled.*

**Table S8. Third-party compensation and punishment scale with moral severity**

| ${Judgment severity}_{i,t}= \beta_{0}+ \beta_{1}{Moral severity}_{i, t} \times\beta_{2}{Judgment type}_{i, t}+\varepsilon$ | | | |  |
| --- | --- | --- | --- | --- |
| Dependent variable | Estimate (SE) | t | p | |
| Judgment severity |  |  |  | |
| Intercept | 14.01 (1.12) | 12.52 | <.001*** | |
| Moral severity | 13.17 (0.65) | 20.18 | <.001*** | |
| Judgment type | 7.22 (0.95) | 7.61 | <.001*** | |
| Moral severity × Judgment type | 2.49 (0.62) | 4.00 | <.001*** | |

| *Note.* Judgment severity ~ Moral severity × Judgment type, where Judgment severity is a continuous variable ranging from -50 to 50. Moral severity and judgment type are indexed by participant and trial. Moral severity is a continuous variable ranging from -50 (not severe) to 50 (very severe) and has been standardized before being entered into the regression. Compensation serves as the reference (0) for Judgment Type. The random effects structure is maximal.  *** p <.001 |
| --- |
|  |

# 4 Experiment 4

## 4.1 Methods

**Experimental design overview**. In Experiment 4, participants selected partners for both the trust game (TG) and dictator game (DG). Here, we report the data for DG partner selection (TG is reported in the manuscript).

**Task instructions**. The instructions participants were given depended on whether they were in the victim condition or third-party condition. Participants were given example trials and comprehension questions to ensure they understood the task.

*Instructions for Experiment 3 (victim condition)^[[1]](#footnote-1)^**

*The purpose of this task is to study how people make decisions. You will be making real decisions that affect the monetary outcomes of YOURSELF. You will be playing multiple rounds of this game.*

*You are Player B.*

*In this scenario, Player A has been allotted $1.00. Each Player A (you will play with a different person on each round) has already decided how much of their $1 to share with you. For example, Player A can decide to:*

*1. keep $.90 and give $.10*

*2. keep $.80 and give $.20*

*3. keep $.70 and give $.30*

*4. keep $.60 and give $.40*

*5. keep $.50 and give $.50*

*After observing Player A make a split, you will be asked to determine the monetary outcome of both Player A and yourself. You decide how much money Player A and you get. You can decide to either:*

*1. Reverse: Reversing the proposed split (thereby punishing Player A for an unfair offer and compensating yourself)*

*2. Compensate: Increase your money to equal Player A's money (thereby compensating them for receiving an unfair offer)*

*3. Accept: Agree to the proposed split and keep both Player A's and your money the same*

*4. Punish: Decrease Player A's money to equal your money (thereby punishing Player A for an unfair offer)*

*5. Equity: Equally split the money between Player A and yourself.*

*6. Reject: Reject the offer and decrease both Player A's and your money to zero.*

*Ultimately, you will decide how much money Player A and you actually receive.*

*Dictator Game Instructions*

*In this part of the study, you will see what other mTurk workers chose in the previous task (in Part 1) you just completed. Based on their decisions, you can choose who your partner will be in a new game. This game is described below:*

*Game 1*

*In Game 1, you will have the opportunity to make money by participating in multiple interactions with different partners. The partner will have been given $1 dollar and can decide how much (if any) to give you. Based on their decisions, the money will be distributed accordingly.*

*For example, if your partner chooses to share $.20, you will receive $.20 and they will keep $.80.*

*Trust Game Instructions*

*In this part of the study, you will again see what other mTurk workers chose in the task in Part 1. Based on their decisions, you can choose who your partner will be in a new game. This new game is described below:*

*Game 2*

*In Game 2, you will have another opportunity to make money by participating in multiple interactions with different partners. For each interaction, you will choose how much money to share with your partner. Based on your and your partner's decisions, the money will be distributed accordingly.*

*Prior to beginning the task, we will give you $1. In each interaction, you can choose how much of this $1 (if any) to share with your partner. The money that you decide to share will be quadrupled. For example, if you choose to share $.80, it will turn into $3.20, etc. Your partner can then choose to:*

*1. Share back half of their money with you (in which case you will make more money than you would have if you had kept it all).*

*2. Keep all of the money you sent, leaving you with the portion you chose not to share.*

## 4.2 Results

***Figure S7. Prosocial partners are preferred over antisocial partners for the Dictator Game.*** *Partner preference is computed as the frequency an option is selected from all available trials, such that each option’s endorsement rate is out of 100%.* ***(a)*** *Preference for partners who chose options in the JG as the Victim.* ***(b)*** *Preference for partners who chose options in the JG as a Third-Party.* ***(C)*** *Partner preference for prosocial (Compensate, Equity, & Accept) and antisocial (Punish, Reject, & Reverse) options as a function of deciding as a Victim or Third-Party. Error bars are ±1 SEM.*

*Partner Selection for Dictator Game by Role (Victim versus Third-Party)*

We see a similar pattern of results emerge for the Dictator Game as was observed in the Trust Game (see manuscript): when potential partners selected responses as the victim, participants showed a strong preference for engaging with those who responded prosocially and not punitively (Friedman’s test, χ^2^(1) = 88.36, *p* < .001, Wilcoxon Signed-Rank test post-hoc effect size r = .91; Fig S7a). Participants least preferred partners who “rejected” offers in previous JGs (12%) and most preferred partners who “compensated” in the wake of a fairness violation (81%). These findings illustrate that there are strong reputational benefits associated with being a victim who “turns the other cheek” in response to being treated unfairly. When potential partners responded as a third-party, participants still showed a preference to select those who were prosocial (i.e., non-punitive; Friedman’s test, χ^2^(1) = 51.84, *p* < .001, Wilcoxon Signed-Rank test post-hoc effect size r = .75; Fig S7b). Again, participants least preferred potential partners who “rejected” offers in previous JGs (14%) and most preferred partners who “compensated” (80%).

*Preference for antisocial third-parties but not antisocial victims as partners in the Dictator Game*

When directly comparing preferences for those who responded antisocially as a victim versus a third-party, we observed a stark difference in how punitive responses shape subsequent partner selection in the Dictator Game, mirroring the findings from the Trust Game (see manuscript). Partners who chose the antisocial, punitive option as a third-party were more likely to be selected as partners than those who responded punitively as the victim (Welch’s *t*-test, *t*(166.7) = 6.04, *p* < .001, g = .85; Fig. S7c).

*Contextual effects of Trust Game and Dictator Game*

We had participants choose partners for both the Trust Game and Dictator Game in order to explore the reputational benefits associated with punishing across two different social contexts—generosity (as indexed by the DG) and trust (as indexed by the TG). To test whether choosing a partner for a trustworthy or generous task (TG vs. DG) influenced participants’ behavior, we conducted a paired-sample t-tests that examined the endorsement rates between the Trust and Dictator Games (see Fig. S8 for all data). All p-values survive Bonferroni corrections for 6 comparisons for victims and 6 comparisons for third-parties.


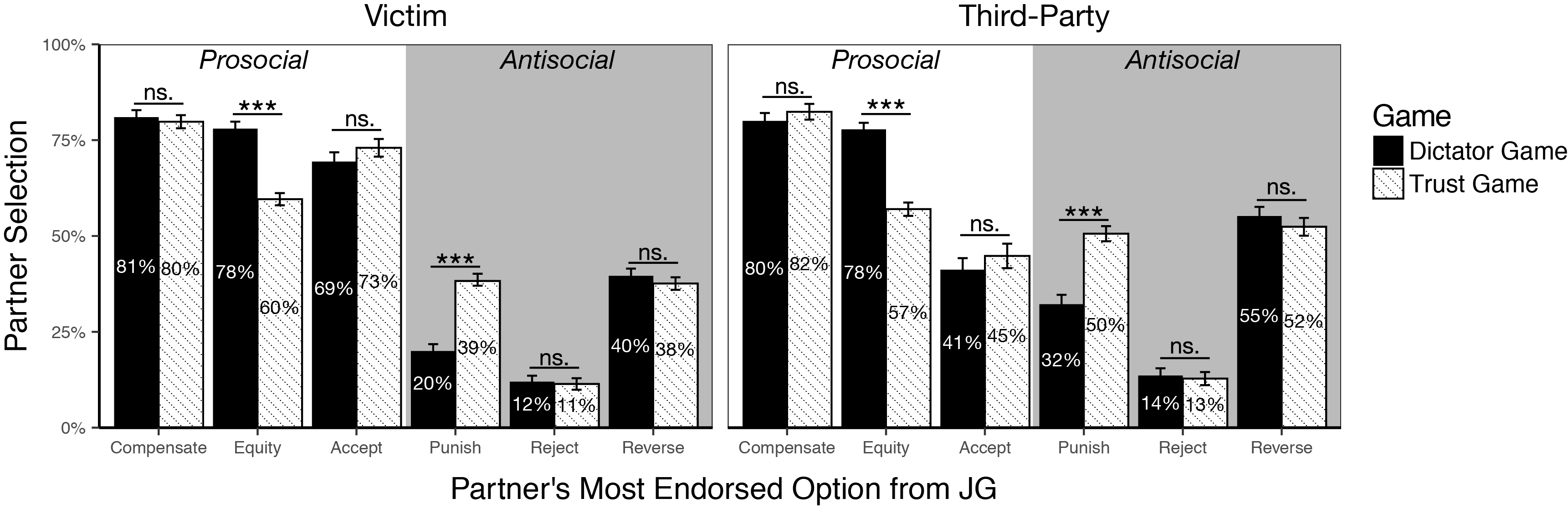


***Figure S8. Reputation Effects of Punitive & Non-Punitive Redistributions Indexed by Role and Game. A)*** *preference for partners who selected options in the JG as the victim for both the TG and DG.* ***B)*** *preference for partners who selected options in the JG as a third-party for both the TG and DG. Error bars are ±1 SEM.*

Those who chose the equitable option in previous Justice Games are more often selected as partners in the DG compared to the TG—both when the decision was made as the victim (paired *t*-test, *t*(99) = 11.32, *p* < .001, g = 1.07) and when the decision was made as a third-party member (paired *t*-test, *t*(99) = 11.88, *p* < .001, g = 1.20). This suggests that those who select the equitable option in the JG exhibit strong inequality aversion^2^, which may be taken as an indication that they will make an equal split when playing as the Proposer in the DG.

In contrast, those who selected the punish option in previous Justice Games are more preferred as partners in the TG, both when the decision was made as the victim (paired *t*-test, *t*(99) = 9.68, *p* < .001, g = 1.09) and when the decision was made as a third-party member (paired *t*-test, *t*(99) = 8.11, *p* < .001, g = .82). These results not only accord with previous work (Jordan, Hoffman, Bloom, & Rand, 2016), but they also illustrate that the social norms of reciprocity are stronger in the TG compared to the DG^3^. In other words, choosing to enforce a social norm by punishing in the JG seems to act as a signal of willingness to follow normative reciprocal behavioral patterns.

# 5 Experiment 5

## 5.1 Methods

**Experimental design overview**. In Experiment 5, participants were presented with a detailed crime vignette to test the reputational effects of a victim or third-party deciding to punish (or not punish) a perpetrator. Using a between-subjects design, participants read one of four vignettes listed below.

**Hypothetical Vignette**. Vignettes 1 and 2 describe the victim punishing (**in bold**) or the victim not punishing (*in italics*). Vignettes 3 and 4 describe the third-party punishing (**in bold**) or the third-party not punishing (*in italics*).

**Vignette 1 & 2: Victim punishes** *[or Victim does not punish]*

Ann is the owner of a clothing store. For a few weeks, she has been watching a woman come into her store who looks around but always leaves without making a purchase. Ann suspects that the woman is shoplifting, which is confirmed when Ann sees the woman stealing some clothing items. Ann goes to confront the woman, but the woman runs out before being caught.
  
The next day, Ann is walking around town when she suddenly sees the same woman in a different store. Ann observes the woman going into a dressing room to try on some clothes and the woman leaves her purse just outside the curtain. Ann realizes she could take the money from the purse as compensation for the stolen clothes. **Ann decides to take the wallet to punish the woman for shoplifting.** *[or: Ann decides to leave the wallet and not punish the woman for shoplifting.]*

**Vignette 3 & 4: Judge punishes** *[or Judge does not punish]*

Ann is the owner of a clothing store. For a few weeks, she has been watching a woman come into her store who looks around but always leaves without making a purchase. Ann suspects that the woman is shoplifting, which is confirmed when Ann sees the woman stealing some clothing items. Ann goes to confront the woman, but the woman runs out before being caught.
  
The next day, Ann is walking around town when she suddenly sees the same woman in a different store. Ann calls the police and they come arrest the woman for shoplifting. **In the courtroom, the judge punishes the woman by imposing a fine for shoplifting.** *[or: In the courtroom, the judge does not punish the woman by imposing a fine for shoplifting.]*

**Task Instructions**.

*Instructions for Experiment 5*

*The purpose of this task to is understand your perceptions of crime. 

You will read a short description of a crime and make a judgment. Afterward, you will answer short demographic survey and finish the study. 

PLEASE READ THE DESCRIPTIONS CAREFULLY.*

# References

1 U.S. Department of Justice, F. B. o. I. *Uniform crime reporting handbook : UCR*. (Revised 2004. [Washington, D.C.] : U.S. Dept. of Justice, Federal Bureau of Investigation, 2004., 2004).

2 Fehr, E. & Schmidt, K. M. A Theory of Fairness, Competition, and Cooperation. *The Quarterly Journal of Economics* **114**, 817-868 (1999).

3 Camerer, C. F. Behavioural studies of strategic thinking in games. *Trends Cogn Sci* **7**, 225-231 (2003).

1. * Third-party condition’s instructions were altered to indicate that they were playing as a third-party (Player C) on behalf of an anonymous Player B. [↑](#footnote-ref-1)
